# Signatures of micropeptides encoded by lncRNAs in cancer progression and metastasis

**DOI:** 10.1038/s41416-026-03556-1

**Published:** 2026-07-27

**Authors:** Stav Zok, Michal Linial

**Affiliations:** 1 The Rachel and Selim Benin School of Computer Science and Engineering, The Hebrew University of Jerusalem, Jerusalem, Israel; 2 Department of Biological Chemistry, The Life Science Institute, The Hebrew University of Jerusalem, Jerusalem, Israel

**Keywords:** Cancer genomics, Data integration, Gene expression profiling

## Abstract

**Background:**

Long non-coding RNAs (lncRNAs) regulate gene expression, chromatin organization, and cellular signaling. Although traditionally considered non-coding, 21% of the ~190,000 annotated lncRNA transcripts contain poorly characterized open reading frames with unknown function.

**Methods:**

We systematically identified lncRNAs encoding micropeptides (MPs) using integrated computational and experimental evidence. Expression profiles across 17 cancer types from The Cancer Genome Atlas (TCGA) were analyzed to identify cancer-associated and transitional lncRNAs (Tr-lncRNAs). Structural modeling using AlphaFold was further applied to predict folding.

**Results:**

We identified 478 lncRNA genes encoding 1782 MPs (10–100 amino acids). These MPs exhibit distinct amino acid and dipeptide compositions and are enriched for specific 4-mer motifs compared with canonical proteins. A subset of lncRNAs, including TNN-AS1, PVT1, XIST, and SNHG family members, encode multiple MPs. Analysis across cancer stages identified 2399 Tr-lncRNAs, most of them were cancer type and stage specific. Among these, 314 highly confident MPs from 72 Tr-lncRNAs were further analyzed. Pan-cancer analysis suggested MP-like functions for Tr-lncRNAs such as LINC01234, HAND2-AS1, XIST, UCA1, and HOXA11-AS. While most MPs are predicted to be intrinsically disordered, 3D structural modeling revealed several MPs with stable folds, including ubiquitin-like and RNase H-like structures.

**Conclusions:**

Tr-lncRNA–derived MPs represent a previously underexplored class of potentially functional molecules associated with cancer clinical annotation and may serve as biomarkers for disease progression.

## Background

Cataloging of long non-coding RNAs (lncRNAs) has identified over 35,000 genes and approximately 190,000 transcripts in the annotated human genome [1]. Although lncRNA regulation resembles that of protein-coding mRNAs, including splicing and transcript maturation (e.g., 3′ polyadenylation and 5′ capping), lncRNAs display distinct features. They are generally poorly conserved, expressed at relatively low levels, and predominantly localize to the nucleus and chromatin. Expression analyses further reveal pronounced tissue and cell-type specificity [2].

Advances in proteomics and translation technologies have uncovered micropeptides (MPs) encoded by short open reading frames (sORFs) within lncRNA sequences, typically 10–100 amino acids (aa) in length [3]. Ribosome-profiling (Ribo-seq) and proteogenomic studies suggest that hundreds to several thousand putative MPs may originate from lncRNAs [4]. However, high-confidence, experimentally validated cases remain rare, with strong evidence limited to only dozens of genes [5]. Consequently, both the criteria for defining bona fide coding products from lncRNAs [5] and estimates of how many proteins arise from non-canonical ORFs remain unresolved [4]. Support for lncRNA coding capacity therefore relies on integrated computational and experimental approaches [6, 7]. The poor evolutionary conservation of lncRNAs further limits the effectiveness of cross-species conservation tools, such as PhyloCSF, for identifying functional sORFs [8].

Like canonical proteins, many lncRNA-derived MPs initiate with methionine (Met) and are translated after transcript export to the cytoplasm [9]. Direct detection methods, including antibody-based assays and mass spectrometry (MS), provide the most compelling evidence by identifying peptides directly. Ribo-seq data from mouse cells suggest that nearly half of lncRNAs may contain translated sORFs, whereas MS-based peptidomics combined with RNA-seq in human K562 cells detected only minute quantities of small peptides. Notably, MS analyses showed that many detected sORFs lack methionine as a start codon [10]. PepScore enables genome-wide evaluation of translated ORFs using ribosome profiling data and predicts peptide stability in human cells [11]. In addition, under stress conditions, translation from lncRNAs may occur independently of the 5′ cap or classical ribosome-binding sites, instead utilizing internal ribosome entry sites (IRESs) [12]. Until recently, experimental evidence for lncRNA-derived ORFs was largely confined to isolated examples.

In cancer, some lncRNA-derived MPs function as tumor suppressors, whereas others promote tumor progression by modulating key biological processes. A few dozen lncRNA-encoded MPs (typically 30–100 aa), have been experimentally validated in specific cancer contexts [13], although their biological roles remain incompletely characterized. One example is the HOXB-AS3 MP, a 53-aa peptide that suppresses colon cancer by attenuating aerobic glycolysis through inhibition of hnRNPA1-mediated alternative splicing of pyruvate kinase M (PKM) mRNA, a pathway linked to the Warburg effect and tumor growth. Loss of HOXB-AS3 MP correlates with poor prognosis and enhanced metabolic reprogramming [14]. Conversely, HOXB-AS3 overexpression in oral squamous cell carcinoma (OSCC) is associated with poor prognosis, where it interacts with the RNA-binding protein IGF2BP2 to stabilize the oncogene c-Myc, thereby promoting cancer cell growth [15]. Another example is LINC00992, whose MP GT3-INCP (GATA3-interacting cryptic protein; 120 aa) drives estrogen receptor–positive (ER + ) breast cancer progression by binding the lineage-defining transcription factor GATA3 and facilitating hormone-driven tumor growth, ultimately enabling escape from hormone-dependency therapies [2]. A recent review summarizes several dozen MPs and their cancer-associated functions across diverse cellular contexts [6]. Collectively, these findings suggest that MPs may have clinical utility as diagnostic biomarkers or therapeutic targets [16, 17].

In this study, we aimed to characterize the protein-coding capacity of lncRNAs across major cancer types by integrating Ribo-seq profiling data from SmProt with TCGA datasets [18]. Cancer-specific lncRNA expression patterns highlighted many lncRNAs that show transition-specific differential expression. We focused on lncRNAs whose expression changes significantly across pathological transitions, termed transitional lncRNAs (Tr-lncRNAs). We identified lncRNAs encoding confident MPs supported by statistically significant experimental evidence. We have analyzed the structural features of 1,782 high-confidence MPs derived from 478 lncRNA genes using AlphaFold2.0 predictions [15]. In addition, we focused on pan-cancer analysis that indicates defining features of lncRNA-encoded MPs and highlights their potential roles in cancer diagnosis, prognosis, immune activation, and cell–cell communication during cancer progression and metastasis.

## Methods

### Naming and harmonization

The naming of lncRNAs is often tied to specific catalogs and databases, and major lncRNA resources use non-coherent indexing systems. Key databases, including Lnc2Cancer 3 [19], LncRNADisease [14], TANRIC [20], and LNCipedia [21], were incorporated into the protocol mapping implemented in GeneCaRNA [22]. For consistency, we adopted gene names from GeneCaRNA based on the genomic position of each lncRNA, taking transcriptional directionality into account. To ensure unique mapping, we revisited ambiguous cases and required a minimum of >90% sequence overlap spanning at least 200 nucleotides. For example, the lncRNA CYTOR (ENSG00000222041; HGNC:28717) is also referred to as C2ORF59, LINC00152, MGC4677, and NCRNA00152, and is indexed in lncBook under HSALNG0016642 and HSALNG0016653. While TCGA typically adheres to Ensembl gene identifiers, the presence of multiple aliases and gene versions frequently leads to mapping failures, underscoring the need for standardized lncRNA nomenclature.

### Overall filtering scheme and terminology

To define the general set of lncRNAs from which Tr-lncRNAs were derived, we used curated RNA sequencing (RNA-seq) data from The Cancer Genome Atlas (TCGA). For each cancer type, the dataset included annotated genes encompassing potential lncRNAs according to GENCODE v49 [1]. From this inclusive list, we extracted 13,257 lncRNA genes that were represented in the TCGA RNA-seq data.

We defined a subset of these lncRNAs as transitional lncRNAs (Tr-lncRNAs) through the following filtering process. (i) RNA-seq samples were first filtered to exclude healthy controls and samples from low-prevalence cancer types (*n* < 150; Supplementary Table S1), yielding 9803 samples from 24 cancer types. (ii) These samples were further stratified based on pathological annotations. Samples with pathologically defined tumor stage produced a dataset of 6825 samples spanning 17 cancer types. In parallel, samples with clinical evidence of progression from primary to metastatic disease yielded a dataset of 5715 samples across 16 cancer types. For each dataset, differential expression analysis was performed across consecutive tumor stages and metastatic states to define Tr-lncRNAs as the union of lncRNA genes showing significant expression changes ( | z | > 3). The subset of the significant Tr-lncRNAs compiled from 2399 lncRNA genes. In this analysis we determined 7 transitions (each partitioned to up and down regulation for the transition), and 17 major cancer types. The 2399 were labeled as Tr-lncRNA genes.

To perform micropeptide (MP) analysis, we downloaded the SmProt database [23] (see “Database of micropeptides” in “Methods”) and filtered MPs by requiring both translation initiation site (TIS) *p* values and ribosome footprint enrichment and considering periodicity (Ribo-seq). We demand that both *p* values will be <0.05. This filtering resulted in 1782 MPs, referred to as lncRNA-MPs, originating from 478 unique lncRNA genes. Among these, 72 lncRNA genes overlapped with the Tr-lncRNA set and collectively encoded 314 MPs, which we termed Tr-lncRNA-MPs. Supplementary Table S2 lists an additional 255 lncRNA genes and their associated MP counts (755 MPs in total) from the lncRNA-MP collection that could not be evaluated as Tr-lncRNA-MPs due to mapping inconsistencies.

### Differential expression lncRNAs and clinical data for major cancer samples

RNA sequencing (RNA-seq) data and the associated clinical annotations were obtained from TCGA via the UCSC Xena data portal [18]. The RNA-seq data were generated using the HiSeq platform, employing a poly(A)+ selection protocol and paired-end sequencing strategy. Raw read counts for annotated lncRNAs were extracted and processed, and went through the TOIL pipeline, which included library depth normalization [24]. In TCGA, the reported measures are provided as RSEM (RNA-seq by expectation maximization) for estimating gene and isoform expression levels from the raw data. The normalization is based on log2[expected_counts+1] to avoid biases from exceptionally small numbers.

Three types of labeling were used for the clinical annotations: pathological, T stage and M state. We first filtered the lncRNAs, per transition (e.g., I- > II), to include only lncRNAs that have an expected count value of 1.0 in at least 30% percent of the samples (considering each of the two stages). Then, we used z-scores over the log2(FC) of the means over the log(x + 1) expression values of the lncRNA across samples in two consecutive stages. We confirmed that the log2(FC) approximates a normal distribution (not shown). We defined a lncRNA as Tr-lncRNA if it was expressed in at least one of the 17 major cancer types and showed stage-associated differential expression, defined as |z | > 3, for at least one pair of consecutive pathological stages [25]. For each cancer type and each lncRNA, we computed the z-value by standardizing the difference in mean expression between consecutive stages using the cancer type-specific mean and standard deviation of these stage-to-stage differences across lncRNAs. For the pathology stage categories in addition to I → II, II → III, III → IV, a simplified combination of I and II and III and IV, marked as E → L, for early to late tumor progression was considered.

We analyzed three metastasis-related groupings. Among non-metastatic tumors (M0), we stratified samples by primary tumor size using the T component of TNM system [26]. We defined M0-small (M0s) as T1 ( ≤ 2 cm) and M0-large (M0l) as T2–T4 ( > approximately 2 cm and/or greater local extent), where T2 and above indicates a tumor larger than 2 cm. Second, to capture the metastasis-associated shift, we compared M0-large (M0l) to metastatic tumors (M1). Third, for a simplified progression analysis, we collapsed tumors into early (ME) and late (ML) categories (early vs. advanced disease).

### Database of micropeptides (MPs)

The unfiltered comprehensive catalog of lncRNAs was reported from GENCODE V.49 [1]. For harmonization of annotations and unified naming, GeneCaRNA database [22] was used. Altogether, the GeneCaRNA reported on ~220,000 unique ncRNA identifiers, and a gene-centric view report on >22k genes. As many as 20% of these genes are associated with MPs as determined by lncBook 2.0 [27] via GeneCaRNA catalog [22]. Revisiting the unfiltered collection of lncRNAs was needed to retain only the highly confident MPs for downstream analysis.

Small peptides and proteins, also called small ORF-encoded peptides (sORFs) or small encoded peptides (SEPs), were obtained from the SmProt dataset [23], which specializes in small proteins supported by Ribo-seq data across human tissues and cell lines. The dataset initially contained 19,920 different MPs whose peptide length is <100 aa, originating in 2935 lncRNA genes. These MPs were filtered to include only MPs whose translation initiation site (TIS) p-value and Ribo *p* value (ribosome footprint enrichment and periodicity) are <0.05 each. We also reduced redundancy by removing duplicates originating from multiple transcripts of the same lncRNA gene. Altogether, we report on 1782 peptides. Among them, there are 314 peptides assigned to Tr-lncRNAs.

### SignalP 6.0

The prediction of the signal peptide sequence relies on the properties of the signal peptides (SPs) [28]. SignalP predictor combines sequence-based features from the N’-terminal. SignalP provides a likelihood score based on 1–5 positively charged residues (Arg and Lys), hydrophobic core (with tracks of Leu, Ile, Val, and Phe), and the position of a cleavage site (CS), which includes small, neutral residues for SP peptidase. Specifically, the recognition signature for signal peptidase I is based on the presence of Ala, Ser, or Gly at -1 and -3 positions relative to the cleavage site.

### Conservation labeling by lncBook

Human lncRNA gene conservation was based on UCSC genome alignments across 40 vertebrate species from zebrafish to human. The lncRNA homologous sequences are based on the alignment length ( ≥ 50 nt) and a minimal length of >20% of the lncRNA transcripts. LncBook reports on 22,347 human lncRNA genes with some homology. For a specific species, the homologous sequences are determined relative to the Q50 threshold for introns (i.e., median level of intron conservation). A sequence in another species was called homologous if its alignment length and identity exceeded the intronic Q50 threshold [27]. The Q75 conservation score indicates that a lncRNA has higher exon–intron sequence conservation than at least 75% of all annotated lncRNAs. Note that the lncBook conservation scores reflect relative conservation within the lncRNA population, rather than protein-coding-level evolutionary conservation.

### External analysis and bioinformatic tools

For structural prediction, we have used AlphaFold 2.0 and the predicted Local Distance Difference Test (pLDDT) [29]. We also applied the SwissModel server for functional relatedness of MPs with template search and model build modes [30]. The results from the SwissModel server provide a global model quality estimation (GMQE) to evaluate the quality and accuracy of structural models. A score for homology models is provided with interactive 3D viewers.

The following Python libraries were utilized in the analysis: (i) Matplotlib, for data visualization and plotting. (ii) SciPy (scipy.stats), for performing statistical analyses, including Fisher’s exact test. (iii) Statsmodels, for multiple testing correction using the false discovery rate (FDR) approach. (iv) To reduce redundancy among predicted peptides, we employed CD-HIT (version 4.8.1) to generate UniRef-style non-redundant sets. Input amino acid sequences were clustered at two sequence identity thresholds corresponding to UniRef definitions: 90% (UniRef90) and 60% (UniRef60). Word size was set according to CD-HIT recommendations for each threshold.

## Results

### Most lncRNAs are cancer transition-specific

Long non-coding RNAs (lncRNAs) are broadly defined as non-coding transcripts longer than 200 nucleotides. In cells, lncRNAs act through multiple, non-exclusive mechanisms, including the production of micropeptides (MPs). From a collection of approximately 190,000 lncRNA-associated transcripts (GENCODE v49), we focused on a small subset of lncRNAs that are likely to exert their function via encoded MPs in cancer samples spanning major tumor types in The Cancer Genome Atlas (TCGA). We compiled a set of 1,782 high-confidence lncRNA-derived MPs, originating from 478 lncRNA genes (Supplementary Table S3).

We next focused on lncRNAs that may act during cancer progression and transition. To this end, we integrated publicly available TCGA expression data with lncRNA gene annotations. Genome coordinates were used to ensure unique mapping, resulting in a final set of 13,257 lncRNA genes. For each TCGA cancer sample, clinical pathology annotations were used to define tumor stage–based (T-stage) and metastasis-based (M-state) categories. LncRNAs were analyzed for differential expression, partitioned into up- and downregulated groups, across consecutive clinical stages within each major cancer type. The lncRNAs that were differentially expressed ( | z-score | > 3) and showed consistent behavior across cancer samples, in any stage transition, were defined as transitional lncRNAs (Tr-lncRNAs); [25]. Tumor stage–based transitions included I → II, II → III, and III → IV, as well as a simplified grouping of stages I–II versus III–IV, denoted as early-to-late (E → L) progression. Metastasis-based transitions included non-metastatic tumors stratified by size (small to large; M0s → M0l) and the transition from primary to metastatic disease (M0l → M1). In addition, a simplified metastasis category contrasting early and late states (ME → ML) was included. In total, seven distinct transitions were analyzed, with E → L transitions serving as a coarse-grained partition of cancer progression.

We identified numerous lncRNAs that were uniquely associated with specific cancer types and distinct disease transitions. Supplementary Fig. S1 illustrates Upset plots for the upregulated and downregulated lncRNAs for the E→L transition (marked positive and negative, respectively). Inspection of the plots shows that the vast majority of Tr-lncRNAs are specific to only one cancer type and mostly for one transition [25]. For example, there are 40 of 44 Tr-lncRNAs that are uniquely associated with the downregulation of E→L in UCEC (Supplementary Fig. S1). Our analysis covers 17 cancer types represented in TCGA ( > 150 samples each). Altogether, we identified 2399 Tr-lncRNAs among the 7 transitions tested for T-stage (4) and M-state (3) see “Methods”, [25].

### A small fraction of Tr-lncRNAs encode micropeptides (MPs)

In this study, we focus on the protein-coding capacity of lncRNAs, specifically lncRNA-derived MP (lncRNA-MPs), with emphasis on those represented in cancer samples and, within this group, on transition-specific lncRNAs (Tr-lncRNAs).

Supplementary Fig. S2 shows the fraction of lncRNAs (in %) with MP production capacity across all cancer types without quality filtration, as reported by lncBook (see Methods). Approximately 21% of TCGA lncRNAs are predicted to contain potentially short open reading frames (sORFs). However, this fraction increases markedly among Tr-lncRNAs in specific cancers. For example, in colorectal adenocarcinoma (COAD), lncRNAs producing MPs account for 55% of Tr-lncRNAs exhibiting a positive expression trend along the E → L transition. This enrichment in MP production capacity among Tr-lncRNAs is statistically significant (all cancer types except KIRC show multiple-testing–corrected p-values < 0.05; Supplementary Fig. S2).

**Fig. 1:**
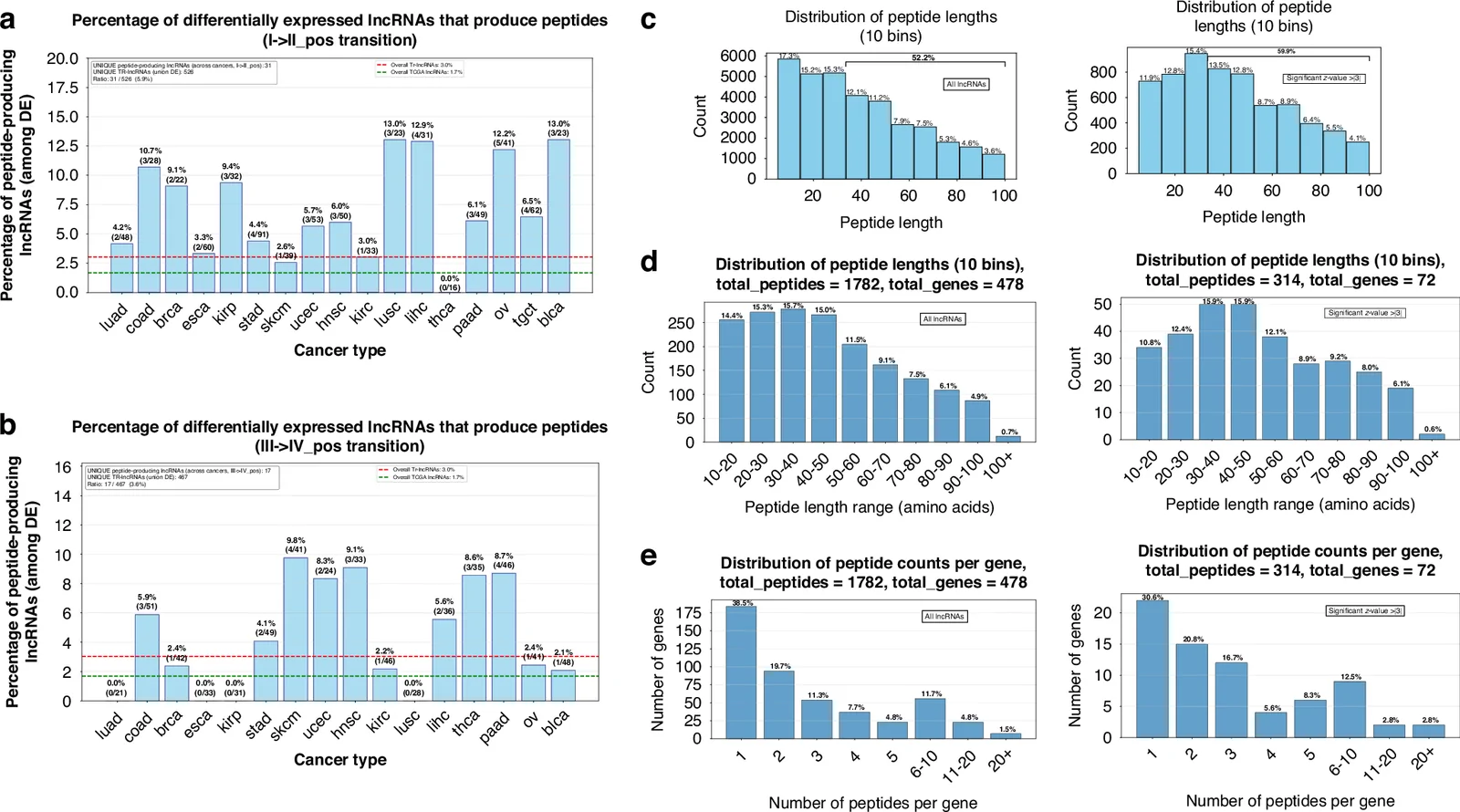
Percentage of MPs-producing lncRNAs among Tr-lncRNAs across cancer types and their properties. **a** Fraction of I → II upregulated lncRNAs among 17 major cancer types (Methods). Only Tr-lncRNAs with confident MPs are considered. Green and red horizontal lines show percentages of lncRNAs producing confident MPs among all lncRNAs and among Tr-lncRNAs, respectively. Numbers of unique MPs per cancer type and overall summary statistics are indicated (top, left). **b** Fraction of III → IV upregulated lncRNAs among 16 major cancer types (TGCT excluded due to missing stage IV samples). Case counts are indicated above bars. Dashed green and red lines indicate percentages of lncRNAs producing confident MPs among all lncRNAs and Tr-lncRNAs, respectively. **c** Peptide length distribution for all lncRNAs, binned in 10 intervals; 52.2% of lncRNA-MPs are ≥30 aa (left). Peptide length distribution for lncRNAs with |z-value | >3; 59.9% are ≥30 aa (right). **d** Length distribution of all confident lncRNA-MPs and Tr-lncRNA-MPs ( | z | >3). Longest peptides are 100 aa. **e** All lncRNAs partitioned by number of MPs produced for Tr-lncRNAs (left) and Tr-lncRNA-MPs ( | z | >3, partitioned by MP number.

We applied strict filters to the sORF collection to exclude MPs lacking sufficient computational and experimental support. Specifically, a confidence threshold based on internal statistics from the SmProt scoring system was applied. SmProt defines high-confidence MPs by combining translation initiation site scores (TIS) with experimental evidence from Ribo-seq (see “Methods”). Figure 1 shows the fraction of Tr-lncRNAs associated with high-confidence MPs across major cancer types in selected transitions. Among all 13,257 lncRNAs, this fraction is 1.7%, whereas among the 2,399 Tr-lncRNAs it is 3.0% (horizontal green and red dashed lines, Fig. 1). For each major cancer type, the bars indicate the percentage of Tr-lncRNAs that produce MPs. Figure 1a, b illustrates MP production among upregulated Tr-lncRNAs across major cancer types along T-stage transitions I → II and III → IV, respectively. Statistical testing for each cancer type was not performed due to the limited number of cases. Still, Fig. 1a indicates five cancer types with >10% occurrence of Tr-lncRNAs with MP capacity (COAD, LUSC, LIHC, KIRC, and OV). Figure 1b shows the analysis for the III → IV transition, where none of the cancer types reach a 10% proportion. Supplementary Table S1 lists TCGA cancer abbreviations and summary statistics.

Comparing the two T-stage transitions across all cancer types, the fraction of lncRNAs with high-confidence MPs is lower in the III → IV transition than in the I → II transition (3.6% vs. 5.9%). Restricting candidate Tr-lncRNA genes to a small, high-confidence MP set enables analysis of the most reliable lncRNAs while maintaining evidence of relevance to cancer progression. Overall, across all transitions, cancer types, and expression directions, we identify 478 lncRNAs producing 1782 peptides ( ≥ 10 amino acids; Supplementary Table S3). All subsequent analyses are based on this stringent gene set and their corresponding MPs.

### The protein capacity of lncRNAs in TCGA

Despite their classification as non-coding, accumulating evidence highlights the role of lncRNA-encoded MPs in oncogenesis [13]. Figure 1c shows the length distribution of all unfiltered lncRNA-MPs, demonstrating the expected inverse relationship between MP length and abundance. In contrast, MPs encoded by lncRNAs involved in cancer transitions (Tr-lncRNAs, defined by |z | >3) show a pronounced shift toward longer peptides, with 60% exceeding MPs >30 aa compared with 52% in the full lncRNA-MP set (Fig. 1c, right).

**Fig. 2:**
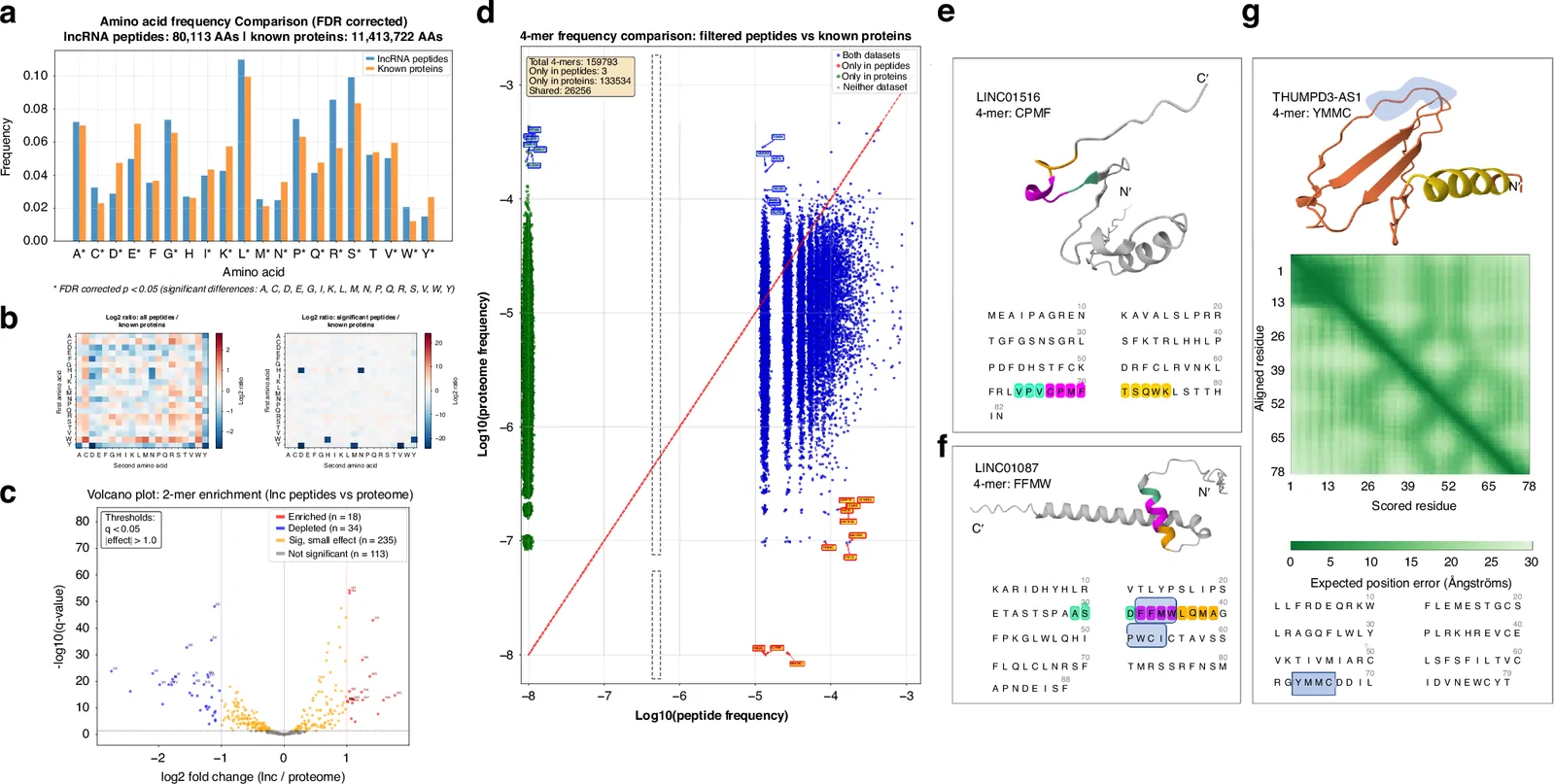
Amino acid (aa) composition. **a** aa distribution in known coding proteins (orange) and high- confidence lncRNA-MPs (blue). Frequency differences for 17 of 20 aa are statistically significant (Fisher’s FDR-corrected <0.05; marked by an asterisk in the legend). The analysis includes 11.4 M aa from known proteins and 80.1k aa from lncRNA-MPs. aa partitions are listed in Supplementary Table S6. **b** Matrix of all 400 dipeptide pairs showing the log2 frequency ratio between lncRNA-MPs (left), TR-lncRNA-MPs (right), and the canonical human proteome. **c** Volcano plot of dipeptide occurrences in lncRNA-MPs versus the human proteome, highlighting significantly enriched (red) and depleted (blue) sets. **d** Analysis of 4-mer sequences in lncRNA-MPs. A scatter plot showing 4-mer frequency in confident lncRNA-MPs relative to the human proteome, clustered into four colored groups. Green marks 4-mers found exclusively in the proteome. Highly abundant 4-mers in either lncRNA-MPs or the proteome are indicated. The x-axis is non-continuous (separated by dashed vertical lines). **e** AF2 structural prediction for the longest MP (82 aa) of LINC01516. The unique 4-mer CPMF is colored magenta. **f** AF2 structural prediction for the longest MP (88 aa) of LINC01087. Enriched 4-mers FFMW and PWCI are abundant in the lncRNA-MP set (blue shading, magenta sequence). pTM = 0.3. **g** AF2 structural prediction for the 79 aa peptide of THUMPD3-AS1. The structure shows low (yellow) and very low (orange) confidence. The unique 4-mer YMMC is shaded blue in the structure and sequence (bottom). The heatmap shows expected positional error (Å) between residue pairs. Darker green indicates higher confidence, and darker regions outside the diagonal reflect local contacts. Overall model confidence is low–medium (pTM = 0.24). The marked 4-mer is predicted to be exposed and not contacting other segments.

Repeating the length analysis using the stringent set of 1,782 high-confidence MPs derived from 478 lncRNA genes shows that, although lncRNA genes with confident MPs represent only 1.7% of all lncRNAs (compared with 21% in the unfiltered set; Supplementary Fig. S2), the length distribution is preserved for both all lncRNA-MPs and Tr-lncRNA-MPs (Fig. 2d). Notably, lncRNAs encoding ≥4 MPs are common, comprising 28.8% of all lncRNAs that produce MPs and 32% of Tr-lncRNAs with this capacity (Fig. 1e).

### Only a third of the confident MP candidates start with initiating methionine

Translation of the human proteome is expected to initiate with methionine (Met, M) from the canonical Kozak ribosomal binding site (ATG codon). In the context of sORFs and lncRNA-encoded micropeptides (lncRNA-MPs), translation initiation can also occur at near-cognate start sites. Ribosome profiling (Ribo-seq) data provide evidence for non-ATG initiation events. Single-nucleotide variants of ATG, including TTG, GTG, CTG, AAG, AGG, ACG, ATA, ATT, and ATC, were observed to support translation initiation (Supplementary Fig. S5), consistent with initiation by Leu, Val, Lys, Arg, Thr, or Ile.

Analysis of the confident MP set ( > 10 aa) showed that only ~30% of peptides initiated with Met (Supplementary Fig. S5). Within the Tr-lncRNA-MP subset, approximately half of the peptides initiated with either Met or Leu. Across all analyzed lncRNA-derived peptide sets, Met was the most frequent initiating residue, followed by Leu, which is encoded by two near-cognate start codons. Following redundancy removal by selecting a representative from clusters sharing >90% amino acid sequence identity (see Methods), the fraction of Met-initiated peptides increased to 37.7%. Notably, approximately two-thirds of the non-redundant MP representatives initiated with either Met or Leu (Supplementary Fig. S5). Together, these results indicate that near-cognate initiation is prevalent among lncRNA-derived MPs, with initiation preferentially occurring at Met and Leu, and lower frequencies observed for Lys, Arg, and Thr.

### The amino acid composition of Tr-lncRNAs-MPs differs from that of canonical proteins

The overall enrichment of MPs within Tr-lncRNAs relative to all lncRNAs (Fig. 1) does not, by itself, indicate whether these MPs resemble or differ from those encoded by canonical protein-coding genes. To address this, we examined whether the amino acid (aa) composition of lncRNA-derived MPs differs from that of the human proteome. We found that lncRNA-MPs deviate substantially from the aa composition of protein-coding genes. Of the 20 amino acids, 17 showed statistically significant differences (Fisher’s FDR corrected p-val < 0.05). Arg (R), Pro (P), Cys (C), and Ser (S) were overrepresented in lncRNA-MPs relative to coding proteins, whereas Glu (E) and Asp (D), and to a lesser extent Asn (N) and Gln (Q), were underrepresented. Only His (H), Thr (T), and Phe (F) did not differ significantly between the two groups (Fig. 2a, asterisk; Supplementary Table S4).

To further evaluate aa distribution trends, we compared high-confidence MPs from all TCGA-reported lncRNAs with those originating specifically from Tr-lncRNAs (Supplementary Fig. S3). Tr-lncRNAs show distinct enrichment patterns, including increased Ile (I) and His (H), alongside depletion trends for Tyr (Y), Val (V), Lys (K), and Trp (W). Notably, comparison of the aa composition of high-confidence lncRNA-MPs (80.1k aa) with the unfiltered set containing largely unsupported MPs (1.33 M aa) revealed highly similar profiles (Supplementary Fig. S3). These results indicate that the aa composition of confident lncRNA-MPs closely mirrors that of the full lncRNA-MP collection, despite the lack of experimental validation or statistical support for most candidates (see Methods). A list of all confident lncRNA-MPs (1,782 peptides), as well as the subset of 314 peptides derived from Tr-lncRNAs, is provided in Supplementary Table S3 and Supplementary Table S5, respectively.

We next examined dipeptide frequencies (400 possible combinations) relative to the human proteome (11.41 M dipeptides). Figure 2b shows a heatmap of log₂-transformed frequency ratios comparing confident lncRNA-MPs with the human proteome. Positive values indicate enrichment in lncRNA-MPs, whereas negative values indicate depletion relative to known proteins. The strongest enrichment involved combinations containing Trp (W), Arg (R), and to a lesser extent Cys (C). In contrast, depleted residues were dominated by Asp (D), Glu (E), Asn (N), and Tyr (Y). The log₂-transformed frequency ratios for Tr-lncRNA-MPs were even more pronounced (Fig. 2b, right). Statistical significance of dipeptide enrichment and depletion was evaluated using volcano plots (Fig. 2c), with enriched and depleted dipeptides highlighted in red and blue, respectively. Dipeptides meeting stringent criteria (Fisher’s FDR q-value < 1e-10; |FC | > 2) were annotated. Among the most enriched were WR, WL, WC, WM, GR, RG, and RC (18 dipeptides), whereas 34 dipeptides were significantly depleted, including EE, EK, DE, YD, and YA.

To quantify effect size, we applied Cohen’s h to measure differences in dipeptide composition between lncRNA-MPs and the human proteome (Supplementary Fig. S4). Across all amino acid pairs, effect sizes were small (Cohen’s h < 0.07), indicating modest overall compositional differences. Comparison of dipeptide occurrences between lncRNA-MPs and Tr-lncRNA-MPs showed no statistically significant differences. Consistent with these results, Supplementary Fig. S4 confirms that the overall dipeptide effect size separating unfiltered lncRNA-MPs from the human proteome is small. Although lncRNA-MPs show subtle deviations from global proteomic dipeptide patterns (Fig. 2b), no significant differences distinguish Tr-lncRNA-derived MPs from the broader lncRNA-MP set.

Together, these findings indicate that key molecular features—including initiation residues, peptide length, and 1-mer and 2-mer compositions (Fig. 2), are similar between lncRNA-MP and Tr-lncRNA-MP groups but clearly distinct from the canonical proteome.

### Analysis of 4-mer amino acids revealed unique and highly enriched 4-mer sequences among lncRNA-MPs

Based on the dipeptide ratios (Fig. 2c), we asked whether longer motifs such as 4-mers characterize lncRNAs with high-confidence MPs. Theoretically, for 4-mer there could be 160k possible combinations (20^4). Altogether, there are 1.34 M aa in all unfiltered MPs across all lncRNAs. We focused on 4-mers that are unique to the high-confidence MPs within the lncRNA set. Figure 2d and Supplementary Table S6 show the occurrence of 4-mers according to their uniqueness in lncRNA-MPs, as well as a subset of 4-mers that are particularly abundant in this group (Supplementary Table S6). Several classes of 4-mers are not discussed further: (i) extremely abundant 4-mers in the human proteome that are not represented in lncRNA-MPs (Fig. 2d, green symbols), and (ii) 4-mers that are highly abundant in the proteome but rare among lncRNA-MPs (Fig. 2d; e.g., KKKK, TGEK; blue background).

Despite the fact that the human proteome spans 11.41 M aa, whereas lncRNA-MPs comprise only 80.1k aa (0.7% of the human proteome), three 4-mers (IMQC, CPMF, MWNC) were found to be unique to lncRNA-MPs (Fig. 2d, red symbols). Under a simplified estimation, the occurrence of three unique 4-mers in such a limited collection is extremely unlikely (estimated *p* value = 8.3e–75). Figure 2e illustrates the predicted structure of an lncRNA-MPs containing the unique 4-mer CPMF, identified in LINC01516, a poorly characterized lncRNA with an exceptionally long transcript (8 exons, multiple isoforms). Structural prediction using AlphaFold 2.0 (AF2) indicated substantial uncertainty in the folding of this peptide.

An additional set of eight 4-mers was found to be highly abundant among lncRNA-MPs while being rare in the human proteome (Fig. 2d, yellow background). Figure 2f shows one such 4-mer (FFMW) occurring in LINC01087 (88 aa; also known as estrogen receptor-α–regulated long noncoding RNA 1). LINC01087 is an estrogen-responsive gene whose transcription is strongly up-regulated by ERα signaling and is associated with the ER⁺ breast cancer subtype. This 4-mer is part of an α-helix, and the full peptide exhibits intermediate structural confidence (AF2 pTM = 0.3). Figure 2g presents another notable example, the 4-mer YMMC, which appears in multiple peptides encoded by the THUMPD3-AS1 gene (also known as SETD5-AS1; genomic locus Chr3p25.3). THUMPD3-AS1 has been implicated in several cancer types, suggesting a potential oncogenic role [31]. However, existing literature primarily attributes its function to competing endogenous RNA (ceRNA) activity, particularly through miRNA sponging mechanisms involved in tumor progression.

### A set of lncRNAs represented by a large set of candidate peptides

A strict selection of confident MPs from TCGA-reported lncRNAs was essential to ensure their relevance and to better characterize their properties. Even within the small set of identified 4-mers (Fig. 2), we observed that high-occurrence sequences are frequently derived from the same lncRNA gene. For example, LINC01087 was associated with 25 distinct peptides in the unfiltered lncRNA-MP collection (Fig. 2f). In another example, for the enriched 4-mer CMTM, all 11 peptides containing this sequence originated from SLC9A3-AS1. This lncRNA is located within an exceptionally active genomic region ( ~ 10k nt) that is densely populated with lncRNAs exhibiting peptide-coding potential.

We next focused on the top-ranked lncRNAs characterized by the highest number of MPs per gene. Examination of the top 10 such lncRNAs (Table 1) revealed that the majority encode relatively long peptides ( > 30 aa). These lncRNAs originate from comparatively long genomic regions (with substantial variability) and have long transcripts, with an average length of 4,800 nt. Although a total of 1782 peptides were identified across all analyzed lncRNAs, the 10 lncRNAs most enriched in MPs (Table 1) collectively account for 16.7% of all lncRNA-MPs. Notably, TTN-AS1 represents an extreme case, with 55 of its 60 MPs exceeding 30 aa in length (Table 1).

**Table 1:** Lists the top 10 Tr-lncRNA-MPs that carry a high number of MPs per gene, along with their genomic and transcriptomic features.

GeneID (Ensembl)	Gene symbol	Genomic size (kb)	Transcript (nt)	Max Length (aa)	# of MP > 10 aa	# of MP > 30 aa
ENSG00000237298.10	TTN-AS1	258	6297	98	60	55
ENSG00000226688.7	ENTPD1-AS1	357	5112	99	35	22
ENSG00000245694.10	CRNDE	86	6325	75	35	29
ENSG00000247828.8	TMEM161B-AS1	170	15,798	74	28	12
ENSG00000249859.11	PVT1	393	2149	91	27	18
ENSG00000196756.13	SNHG17	15	2261	83	22	17
ENSG00000257621.8	PSMA3-AS1	160	2475	84	22	19
ENSG00000242125.3	SNHG3	12	2614	60	20	12
ENSG00000255717.7	SNHG1	4	2812	66	20	12
ENSG00000233461.6	AL445524.1	7.5	2172	83	19	18

It is important to note that several genes listed in Table 1 have been previously studied in the context of lncRNA functions in cancer, including cancer identity (e.g., PVT1), immunomodulation (e.g., ENTPD1-AS1), and proliferation (e.g., TTN-AS1), among others. TTN-AS1 is represented by 60 individual peptides (Table 1) and constitutes a major Tr-lncRNA associated with the HNSC T-stage E → L transition.

**Fig. 3:**
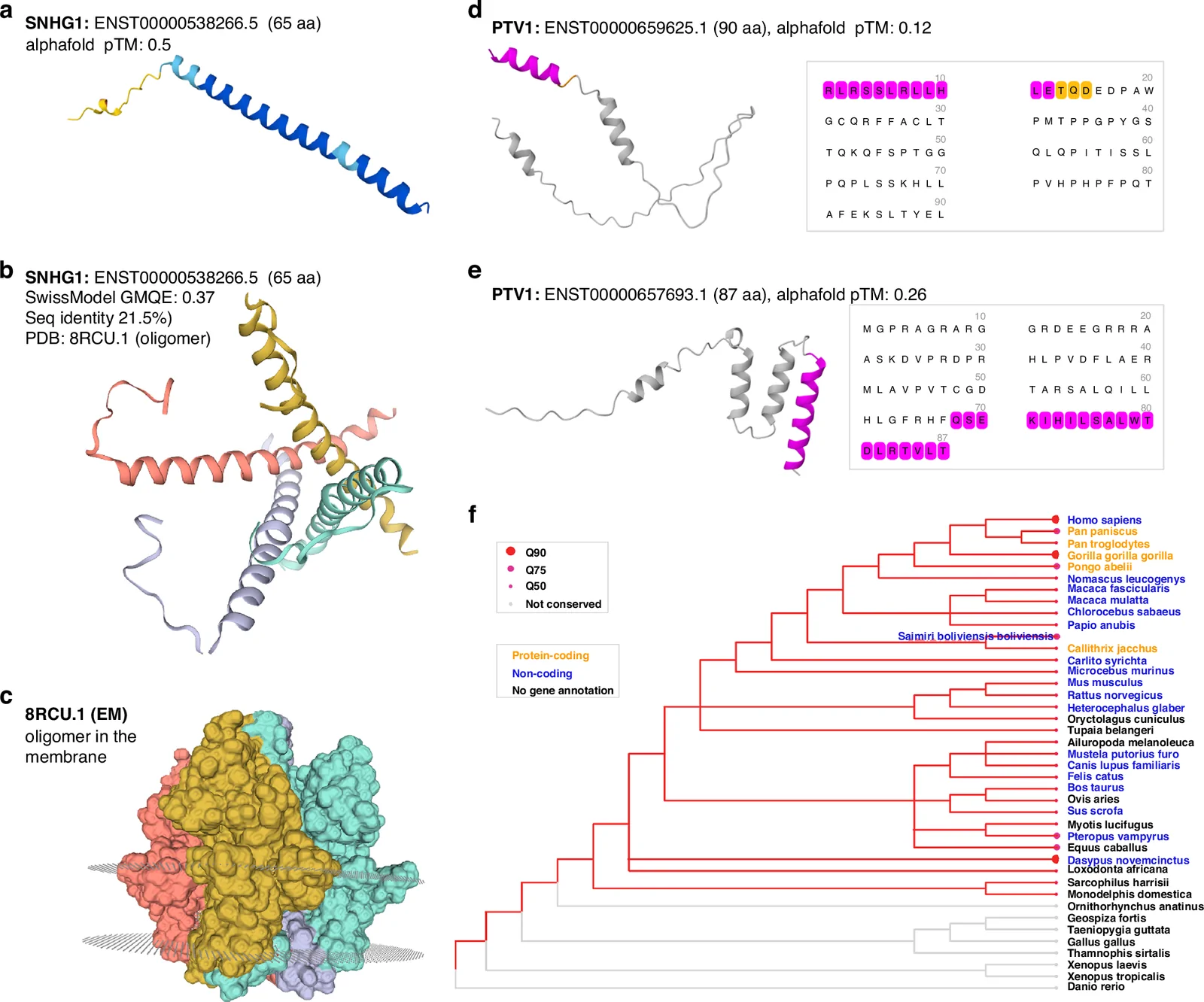
Structure and phylogenetic analysis of lncRNAs with many MPs. **a** Structural prediction of the SNHG1 sequence (65 aa) by AlphaFold2 (AF2). The model shows a very long α-helix with relatively high confidence (pTM = 0.5). **b** SwissModel builds a tetramer model (as in A) positioned to form a conducting channel. **c** The human TRPM4 complex (PDB: 8RCU.1) was used as a template despite low sequence identity. The model is based on the cryo-EM structure of TRPM4 oligomers embedded in a membrane. TRPM4 is a Ca²⁺-activated monovalent cation channel involved in cellular excitability and signal transduction. **d** Sequence of PVT1 transcript variant (90 aa) with low-confidence prediction (pTM = 0.12). **e** Sequence of another PVT1 transcript variant (87 aa) with low–medium confidence (pTM = 0.26). The two transcripts share no sequence overlap. Helical regions shown in magenta correspond to AF2-predicted structures. **f** Phylogenetic tree showing evolutionary conservation of PVT1 homologs across species. Protein-coding homologs are highlighted in orange. The tree shows strong conservation among mammals, with some exceptions. Notably, human lncRNAs correspond to coding homologs in primates.

Among the lncRNAs enriched for MPs, three members of the small nucleolar RNA host gene family (SNHG3, SNHG17 and SNHG1) were identified. These genes are recognized as key drivers of cancer progression. SNHG3 is predominantly upregulated in breast, liver, and lung cancers, whereas SNHG17 shows elevated expression in gastric, colorectal, and lung cancers. The expressed transcripts primarily function as competing endogenous RNAs (ceRNAs) that promote epithelial–mesenchymal transition (EMT), proliferation, and immune evasion. SNHG1 forms a regulatory network that supports its oncogenic role in colorectal cancer and multiple additional cancer types. Figure 3a shows the structural prediction of the SNHG1-derived peptide (65 aa), revealing a long α-helix with very high confidence (blue), while the N-terminal region exhibits lower confidence. Structural modeling using SwissModel yielded a single model suggesting a well-organized tetrameric assembly (Fig. 3b). Inspection of this model indicates that the peptide adopts a membrane-associated structure composed of four coordinated chains, resembling the architecture of the human TRPM4 channel [32]. The TRPM4 tetramer has been resolved by cryo-EM and validated as a cation channel spanning the membrane (Fig. 3c). TRPM4 is a calcium-activated, nonselective monovalent cation channel involved in regulating membrane potential and calcium signaling across diverse biological systems. Based on these observations, we propose that SNHG1-derived lncRNA-MPs may interfere with the function of the native channel by disrupting the oligomeric assembly of TRPM4 [32].

The genes listed in Table 1, including members of the SNHG1 family, have not previously been implicated as acting through their protein products. A notable exception is the PVT1-encoded peptide, which was identified as a tumor antigen in colon cancer [33]. Remarkably, PVT1 spans an exceptionally long genomic region (Table 1) and produces unusually long transcripts (13 exons, with some isoforms reaching 19-20k nucleotides). Several MPs derived from PVT1 do not overlap in sequence, as they originate from distinct exons and alternative ORF usage. Two long, non-overlapping PVT1-derived MPs are illustrated in Fig. 3. AlphaFold2 (AF2) predictions shown in Fig. 3d, e indicate that both peptides are poorly structured, belonging to clusters 1 and 2 (according to Ref60 sequence similarity threshold), respectively (Supplementary Fig. S6; Supplementary Table S7). The predicted structures appear largely unpacked, with low to moderate confidence in folding accuracy. Figure 3f presents a phylogenetic analysis of the PVT1 gene. Notably, PVT1 is annotated as a protein-coding gene in some primates, whereas in humans, gibbons, macaques, and other Old World monkeys it is classified as an lncRNA. Interestingly, in *Callithrix*, a genus of New World monkeys, PVT1 is annotated as a coding gene. Homologs were not detected in treeshrews, rabbits, and several other mammals, highlighting the evolutionary diversity of the PVT1 locus.

**Fig. 4:**
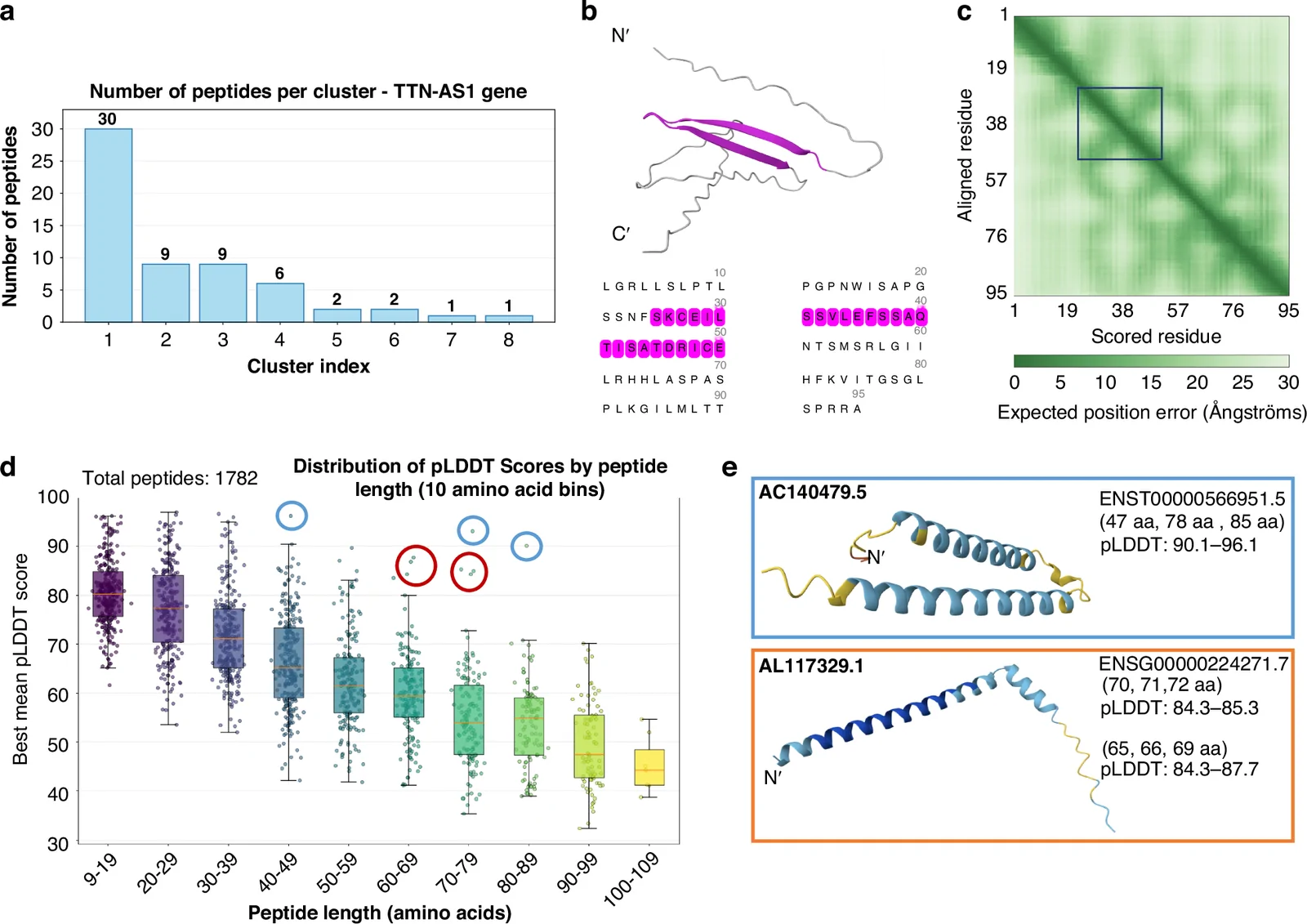
Structural prediction analysis. **a** Clustering of TTN-AS1 Tr-lncRNA MPs by sequence identity ( > 60%). The 8 clusters comprise 60 MPs. **b** Structural prediction of TTN-AS1 Tr-lncRNAs for the longest MP (cluster 1 in A) using AlphaFold2. TTN-AS1 is represented by 60 peptides. Sequence of the 95 aa MP is shown (β-sheets above). TTN-AS1 is a Tr-lncRNA specialized in the HNSC E → L transition. **c** Heatmap of expected positional error (Å) between residue pairs; darker green indicates higher confidence. Darker regions outside the diagonal reflect local contacts, while lighter regions indicate greater uncertainty. Overall model confidence is low (pTM = 0.14). **d** Box plot of AlphaFold2 (AF2) pLDDT by MP length bins. Longer MPs show lower mean pLDDT on average. Red circles mark six MPs (60-79 aa) with high pLDDT assigned to lncRNA AL117329.1, while blue circles correspond to AC140479.5 with very high pLDDT (0.91). **e** AF2 predictions for AC140479.5 (85 aa, blue circles in D) and AL117329.1 (69 aa, red circles in **d**). Representative MP cluster sequences form relatively long α-helices. Bluish coloring of the 3D structures indicates high-confidence pLDDT predictions.

Figure 4a presents representative MPs from TTN-AS1, a prominent Tr-lncRNA implicated in the T-stage E → L transition in HNSC. Figure 4b shows the AF2-predicted structure of a representative TTN-AS1 MP, which consists of multiple β-strands with substantial inter-strand contacts, along with a long intrinsically disordered tail. Figure 4c depicts the estimated positional error between all residue pairs. Overall, the model exhibits low confidence in residue placement (pTM = 0.14), indicating limited reliability of the predicted spatial arrangement. The accompanying heatmap illustrates the expected pairwise positional error (Å) between residues, with darker green regions beyond the diagonal reflecting greater uncertainty. We next evaluated whether pLDDT, which is AF2 per-residue confidence score, could serve as an indicator of MP structural reliability. To this end, we analyzed the relationship between pLDDT values and peptide length (Fig. 4d). Supplementary Table S3 lists all 1782 peptides along with their associated statistical metrics, including AF2 confidence scores. An inverse relationship was observed, whereby longer MPs tended to exhibit lower pLDDT values. Examination of MPs with exceptionally high pLDDT scores revealed enrichment for α-helical structures. Notable high-confidence outliers include AC140479.5 (85 aa) (Fig. 4e, blue circles in Fig. 4d) and AL117329.1 (69 aa) (Fig. 4e, red circles in in Fig. 4d). Both lncRNAs remain poorly characterized. However, AC140479.5 has been reported to be differentially expressed in FOXA2 knockout iPSC-derived pancreatic cells, suggesting a potential role in cell identity and organ development [34].

In conclusion, a subset of lncRNA-derived MPs appears to contain foldable short proteins. As such, their products may serve as protein-based signatures in specific cancer contexts and potentially contribute to the modulation of protein-protein interactions.

### Predicted signal peptide sequence highlights MPs of the secretory system

We applied SignalP 6.0 to predict signal peptides (SP, see Methods) in the 1,782 high-confidence MPs. Only 6 MPs were predicted with high confidence to contain a signal peptide, corresponding to four unique lncRNA genes: ZNF433-AS1, AC020928.1, LOXL1-AS1, and SNHG17 (Supplementary Table S3; Supplementary Fig. S7). In all 4 cases, a clear cleavage site was predicted around residues 20–30 aa, suggesting that these MPs may be secreted or targeted to the endoplasmic reticulum. LOXL1-AS1 is an antisense transcript of LOXL1, which plays a role in extracellular matrix remodeling and fibrosis through collagen and elastin crosslinking. ZNF433-AS1 remains poorly characterized but may influence the expression of ZNF433, a gene involved in chromatin remodeling. SNHG17, similar to other members of the SNHG family, has been implicated in cancer development and progression. Collectively, these results indicate that only a negligible fraction of lncRNA-derived MPs exhibit classical signal peptide features.

### Overlooked Tr-lncRNA-MPs show similarity to known functional protein domains

**Fig. 5:**
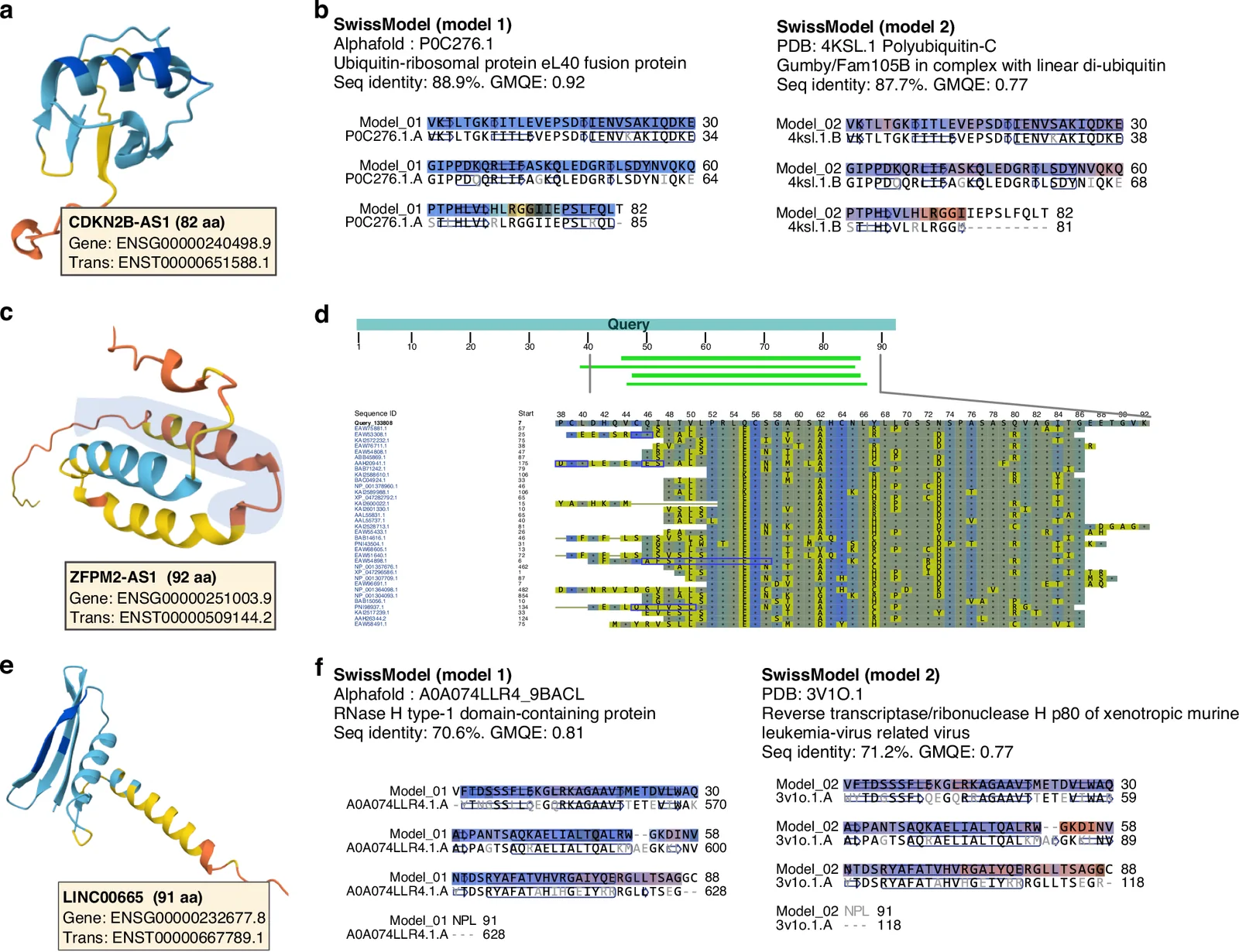
Predicted MPs encoded by selected Tr-lncRNAs. **a** CDKN2B-AS1 (ENSG00000240498.9; transcript ENST0000051588.1; 82 aa). The predicted 3D fold shows mixed α-helical and β-strand elements. **b** Predicted peptide with best structural homologs from SwissModel. The two top models correspond to ubiquitin. Conserved residues are shaded blue, and low-similarity regions are red. **c** ZFPM2-AS1 (ENSG00000251003.9; transcript ENST0000059144.2; 92 aa). The predicted structure contains several well-packed α-helices of unknown function. The shaded helix resembles a short ~40 aa domain, highlighted in light green along the sequence. **d** Multiple sequence alignment (MSA) of the overlapping helical domain. Blue indicates identical aa between the query and homologs; yellow marks consensus residues among homologs. High sequence similarity spans proteins from humans and other primates. **e** LINC00665 (ENSG00000252677.8; transcript ENST0000067789.1; 91 aa). SwissProt predicted models show sequence and structural overlap with an RNase H type-1–like fold. **f** Pairwise alignment with the two top SwissModel models. Similarity to RNase H type-1 domain-containing protein and reverse transcriptase/RNase H is evident. Conserved aa are blue; highly diverse residues are red.

In our analysis, we applied strict thresholds to define MPs and Tr-lncRNAs. In total, we curated a confident set of 1782 high-confidence MPs, of which 314 peptides (from 72 genes) met the Tr-lncRNA criteria and were consistently mapped across the resources used (see Methods). We next examined whether a subset of MPs could be functionally interpreted. To this end, we applied SwissModel to generate likely 3D structural models and AlphaFold2 (AF2) to assess foldability and estimate positional error between amino acid pairs. Figure 5 highlights three Tr-lncRNA-derived MPs with their predicted structures and confidence scores. We used the GMQE score, a normalized metric comparing each query to its best structural template within SwissModel. Structural predictions were generated using AF2 and integrated with SwissModel templates. Figure 5a presents an MP derived from CDKN2B-AS1 that adopts a ubiquitin-like fold, supported by high sequence similarity and elevated GMQE values (Fig. 5b). Notably, CDKN2B-AS1 is transcribed from the reverse strand of CDKN2B, which encodes p15INK4b, a cyclin-dependent kinase inhibitor regulating the G1 → S phase transition through inhibition of CDK4 and CDK6. Figure 5c depicts ZFPM2-AS1 (92 aa), an understudied MP predicted to fold into a compact four-helix bundle. One helix shows strong sequence conservation among primates within an uncharacterized ~40-residue domain (positions 45–85; Fig. 5d). Figure 5e presents LINC00665 (91 aa), whose predicted structure resembles classical RNase H enzymes found in bacteria, viruses, and other organisms (Fig. 5f).

Collectively, these results suggest that MPs associated with cancer progression or metastatic transitions may function by structurally mimicking known small proteins, such as ubiquitin or RNase H, potentially modulating protein–protein interactions during tumor progression.

### Pan-cancer Tr-lncRNAs are enriched with MPs

**Fig. 6:**
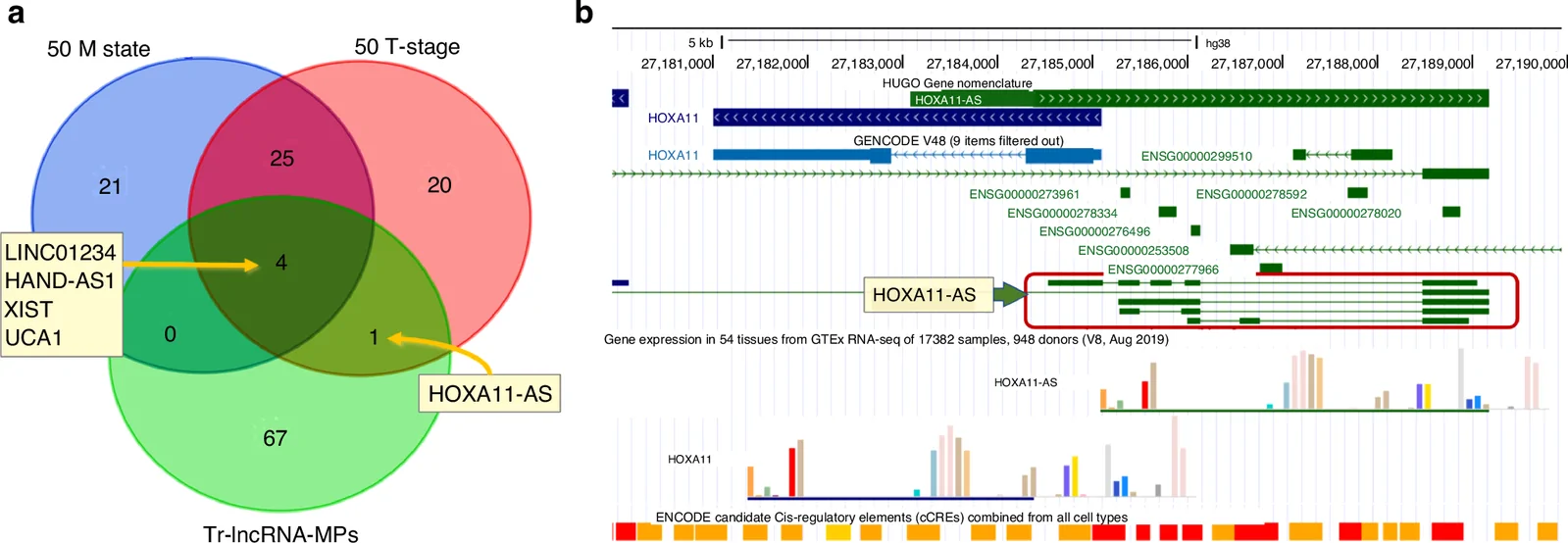
Pan-cancer analysis of Tr-lncRNAs and MPs >30 aa. **a** Venn diagram highlighting shared and unique Tr-lncRNAs assigned as pan-cancer. The top Tr-lncRNAs associated with M-state and T-stage transitions are compared (50 per group). The five genes overlapping between the 72 Tr-lncRNA genes (314 MPs) and the top pan-cancer Tr-lncRNAs are listed. **b** UCSC Genome Browser view of HOXA11 and HOXA11-AS. Tracks span 9,000 nt from Chr7. The HUGO track indicates the overlapping segment of the two genes. GENCODE V48 displays multiple HOXA11-AS transcripts (green). Gene expression across 54 GTEx tissues is shown, with each colored bar representing a tissue. Strong coherence between HOXA11 and HOXA11-AS is observed. The ENCODE track is colored by regulatory element annotations: promoter-like signature (PLS, red), proximal enhancer-like signature (pELS, orange), and distal enhancer-like signature (dELS, yellow).

Pan-cancer analyses reveal that some of the Tr-lncRNAs are shared across multiple cancer types and several of the 7-studied transitions [25]. We investigated whether MPs derived from these top pan-cancer Tr-lncRNAs might contribute to cancer progression. Figure 6 presents a Venn diagram of the top 50 Tr-lncRNAs identified across four T-stage transitions and three M-state transitions in 17 cancer types, regardless of MP presence. We then assessed whether MPs were enriched within these top pan-cancer gene sets. The Venn diagram (Fig. 6a) illustrates the overlap between the T-stage and M-state pan-cancer gene groups (29 shared genes; Supplementary Table S8), representing a highly significant enrichment (hypergeometric test, *p* value < 1e–30). Although the numbers were smaller and did not reach statistical significance, we observed that 5 of the 72 Tr-lncRNAs encoding high-confidence MPs overlapped with the pan-cancer lists (LINC01234, HAND-AS1, XIST, UCA1, and HOXA11-AS).

Figure 6b highlights the dense region encompassing HOXA11 and HOXA11-AS, revealing several genomic features: (i) HOXA11-AS transcripts: Multiple transcript variants are expressed, including an alternatively spliced antisense transcript (red frame). All 5 transcripts are classified as encoding highly confident MPs. (ii) HOXA11 and HOXA11-AS share overlap at the first exon, which contains the HOXA11 start codon (Met). (iii) This genomic locus is transcriptionally active, featuring a cluster of promoters (ENCODE track, red) and enhancer/promoter segments (ENCODE track, orange).(iv) GTEx data show that HOXA11 and HOXA11-AS have highly similar expression profiles, suggesting coordinated transcription. Elevated expression is observed in the cervix, colon, thyroid, tibial artery, bladder, and other tissues. This transcriptional coupling likely extends to the MPs encoded by HOXA11-AS. Among other examined genes (LINC01234, HAND-AS1, XIST, UCA1), only LINC01234 encodes a protein (MBOP) that promotes COAD via MAPK signaling. The remaining genes appear to function primarily through transcriptional regulation and cancer-related signaling effects [6].

## Discussion

This study provides a comprehensive view of the emerging role of lncRNA-encoded MPs and their expression landscape across cancer progression. Although lncRNAs have traditionally been classified as non-coding, growing evidence shows that many harbor sORFs capable of encoding functional peptides [35]. Here, we focused on high-confidence lncRNA-MPs, representing only a small fraction of the large pool of computationally predicted sORFs. Using TCGA data [25], we identified 2399 lncRNA genes differentially expressed across successive stages of cancer progression, termed transitional lncRNAs (Tr-lncRNAs). Transcripts from these genes were enriched for predicted MPs relative to the background set of lncRNA sORFs. Additional high-confidence MPs likely exist but were excluded due to stringent criteria such as minimum sample size and complete clinical annotation. Future work integrating improved detection of non-canonical translation events and additional transcriptomic and proteomic datasets may expand the currently underexplored repertoire of cancer-associated lncRNA-derived MPs [36]. In this study, we restricted analysis to high-confidence MPs uniquely mapped to TCGA samples (478 lncRNAs encoding 1782 peptides from 7.6k samples). Many MPs may remain undetected due to noncanonical features such as alternative start codons, present in over 50% of cases. Additional approaches, including MS and immunosurveillance, may reveal further MPs by identifying novel epitopes [37].

Many lncRNAs encoded multiple peptides. To reduce redundancy, we applied Ref90 and Ref60 clustering (minimum length ≥10 aa), improving the signal-to-noise ratio. Sequence clustering combined with AF2 and SwissModel predictions helped define a hidden peptidome. A correlation between short peptide length and pLDDT scores enabled identification of peptides whose AF2 confidence values deviated from the mean. These peptides frequently formed stable α-helical structures, suggesting potential structural stability and protein–protein interaction capacity.

The average length of lncRNA-MPs was 45 aa, making functional annotation challenging. Longer peptides ( > 80 aa) were more suitable for homology searches and structural inference. Nevertheless, several shorter functional MPs have been described. For example, the 48 aa TUNAR peptide regulates neuronal differentiation and ER calcium homeostasis in pancreatic β cells [38, 39]. A 17 aa peptide from MIR155HG suppresses autoimmune inflammation [40], while a 21 aa peptide derived from MLLT4-AS1 is associated with breast cancer survival [41]. These examples show that very short MPs can exert biological effects, although functional prioritization remains challenging. While most MPs influence intracellular signaling, those containing signal peptides may be secreted and influence the tumor microenvironment (TME). Even peptides lacking signal peptides may be secreted through exosomes [42].

Several lncRNAs have been linked to cancer [43], but most remain unexplored as therapeutic targets. Our analysis highlights cancer-associated lncRNAs, particularly members of the SNHG family. Although SNHG genes are implicated in tumor biology, their MP-encoding potential has been largely overlooked. We identified numerous MPs within SNHG3 (20 peptides), SNHG17 (22 peptides), and SNHG1 (20 peptides). Notably, SNHG17 contains a putative signal peptide that may enable secretion. SNHG genes act as oncogenic drivers across multiple tumor types, including SNHG17 in gastric, colorectal, and lung cancers [44], SNHG3 in breast, liver, and lung cancers, and SNHG1 across several malignancies [45]. Overall, 7.2% of all high-confidence lncRNA-MPs originated from 13 SNHG genes. Several validated lncRNA-derived peptides, including SPAR, HOXB-AS3, and longer MPs such as BXW7 (185 aa) and SHPRH (146 aa), display tumor-suppressive activity [46]. Although our analysis focused on MPs ≤100 aa, HOXB-AS3 emerged as a strong Tr-lncRNA candidate. We also identified LINC01234 as a pan-cancer lncRNA encoding multiple high-confidence MPs. Recently, the LINC01234-encoded peptide MBOP (85 aa) was shown to promote colon cancer cell migration and proliferation through MAPK activation via MEK1 binding and stabilization [47]. Our findings suggest that lncRNA-encoded MPs can contain foldable motifs (e.g., ubiquitin-like or RNase H-related folds), secretion signals, and interaction domains, enabling them to mimic or modulate established protein networks. These peptide activities may complement classical RNA-mediated lncRNA functions such as chromatin organization, scaffolding, and miRNA sponging, reinforcing underappreciated oncogenic mechanisms.

The discovery of antigen repertoires across cancer progression is increasingly relevant for immunotherapy. The oncogenic lncRNA PVT1 encodes a tumor antigen capable of triggering T-cell surveillance [33]. Structural predictions of Tr-lncRNA-MPs revealed that some peptides do not form stable folds and instead display low-confidence helices or largely disordered conformations. Evidence for the protein-coding relevance of PVT1 is further supported by evolutionary conservation. Clinically, high PVT1 expression correlates with poor survival across multiple cancers and is linked to chemoresistance through apoptosis inhibition and altered signaling [48]. Highly expressed lncRNA-MPs in TCGA samples may therefore expand the repertoire of tumor neoantigens. The distinct amino acid, dipeptide, and 4-mer composition of lncRNA-MPs relative to the human proteome likely reflects weaker evolutionary constraints compared with canonical proteins. These features may produce cancer-specific neoantigens that, when presented on MHC class I or II molecules, become visible to the immune system and influence the immune TME [49].

Finally, this study highlights limitations in current genome annotations. Some lncRNAs may represent misclassified protein-coding genes, particularly in cancer contexts. Because lncRNA definitions remain fluid and pseudogenes or incompletely spliced transcripts may contaminate datasets [1], we restricted analysis to reliable lncRNA-MPs curated in SmProt [23]. Complementary resources such as lncBook may further improve MP annotation. However, non-standardized lncRNA nomenclature caused mapping failures and likely underestimation of Tr-lncRNA-MPs, affecting 755 of 1,742 high-quality MPs. Functional inference is also limited by the difficulty of predicting structure for very short peptides ( < 60 aa). Although mass spectrometry provides direct evidence of MP expression, detection remains challenging due to low abundance, limited peptide coverage, post-translational modifications, and context-specific lncRNA expression across cancer stages and sample subsets.

## Conclusions

Our findings show that lncRNA-encoded MPs represent a widespread and previously underappreciated regulatory layer within cancer transcriptomes. By analyzing expression dynamics across cancer progression rather than static tumor profiles, we identified transitional lncRNA-derived MPs (Tr-lncRNA-MPs) likely involved in tumor progression and metastasis. Despite challenges in lncRNA annotation and nomenclature, we report 314 high-confidence MPs and propose potential functions for several, including ubiquitin-like and RNase H–related folds. These peptides may influence processes such as antigen presentation, cell–cell communication, and protein–protein interactions. Further experimental validation will be required to establish their biological and therapeutic relevance in cancer.

## Supplementary information

Summpementary Figures S1-S7

Summpementary Tables S1-S8

## Data Availability

Visualizations and large tables are available on GitHub. https://doi.org/10.5281/zenodo.17170198. Source data for the analysis are provided in the Supplementary materials: Supplementary Figs. S1–S6 and Supplementary Tables S1–S8.
