# Supplementary material for: Signatures of micropeptides encoded by lncRNAs in cancer progression and metastasis
Source: Br J Cancer. 2026 Jul 27;135(9):1390–401. doi: 10.1038/s41416-026-03556-1 (PMC13675225; doi:10.1038/s41416-026-03556-1)
Supplement: Supplementary file 1 [file 41416_2026_3556_MOESM1_ESM.pdf]

Signatures of Micropeptides Encoded by lncRNAs in Cancer Progression and Metastasis

Stav Zok and Michal Linial

Supplementary Materials

Supplementary Tables (in additional XLS file)

Table S1. Abbreviations of cancer types and their occurrences (≥150 samples)

Table S2. Unmapped lncRNAs with MPs that were unmapped in the context of Tr-lncRNAs

Table S3. Detailed Information of 1782 lncRNA-MPs with AF2 and signal peptide analyses

Table S4. Frequencies of aa in lncRNA-MPs (unfiltered) and Human proteome.

Table S5. Detailed Information of 314 Tr-lncRNA-MPs along with annotations

Table S6. Analysis of 4-mer enrichment in lncRNA vs whole human proteome (top). 4-mer exclusive lncRNAs

Table S7. Sequence based clustering of 27 Tr-lncRNA-MPs by sequence similarity (PVT1)

Table S8. The 50 top pancancer Tr-lncRNA for metastasis and for T-stage for each cancer types

Supplementary Figures

Figures S1-S7

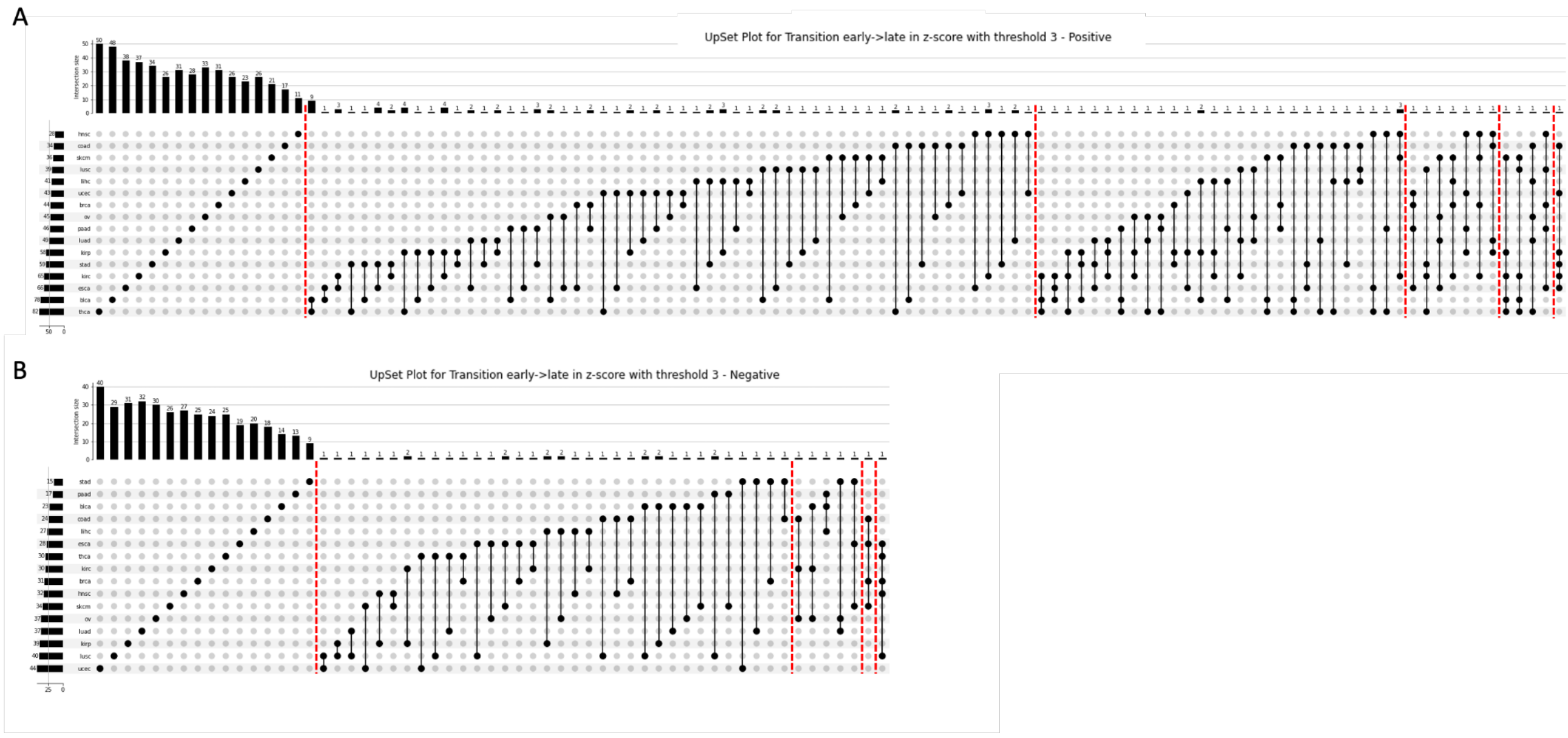

Fig. S1. *Overlapping Tr-lncRNAs across major cancer types. UpSet plots for the intersection and connectivity of Tr-lncRNAs across major cancer types during early (I & II) to late (III & IV) (E→L) transition.* ***(A)*** *Upregulated Tr-lncRNAs (positive) reflect increased expression in the T stage. The dashed red vertical lines group the Tr-lncRNAs by the number of cancer types.* ***(B)*** *Downregulated Tr-lncRNAs (negative) reflect decreased expression in the T stage. All combinations of cancer types are shown. The dashed red vertical lines group the Tr-lncRNAs by the number of cancer types. Horizontal bars (left) summarize the total number of significantly changing lncRNAs in each cancer type (for cancer type abbreviations, see Supplemental* ***Table S1****). Vertical bars (top) count the number of shared lncRNAs across any combination of cancer types.*

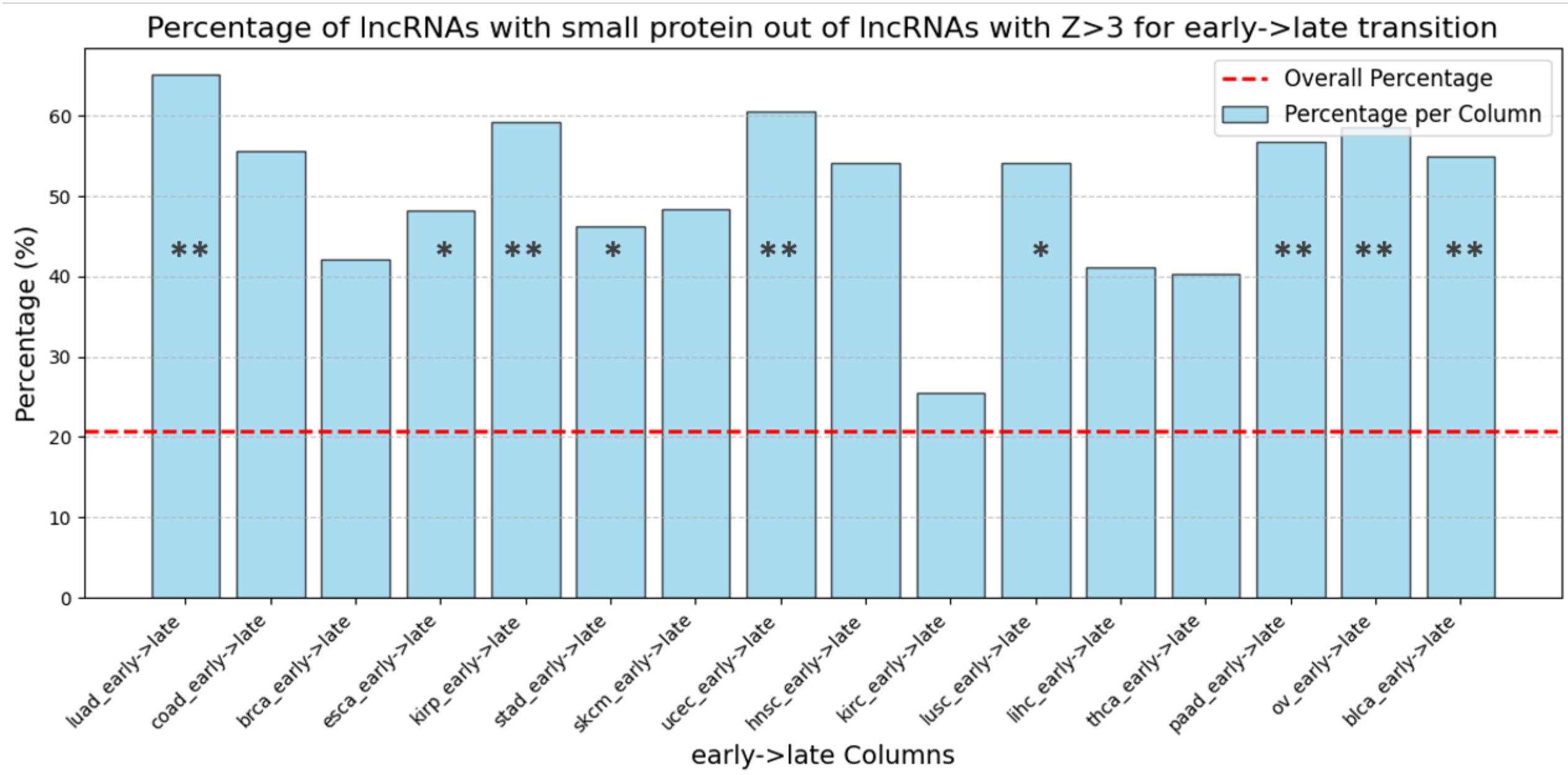

Fig. S2. *The percentage of MPs containing lncRNAs across cancer types. The fraction of E→L transition among major cancer types. Only upregulated sets for E→L transition are shown for any annotated MPs. The dashed horizontal red line reflects the average percentage of MPs containing lncRNAs among all lncRNAs and among all reported sORFs based on lncBook annotations. Corrected p-value <1e-6 and <1e-4 are marked by ** and *, respectively. The enriched fraction of MPs above the unfiltered lncRNAs in each cancer type (excluding KIRC) is significant with a corrected p-value <0.05.*

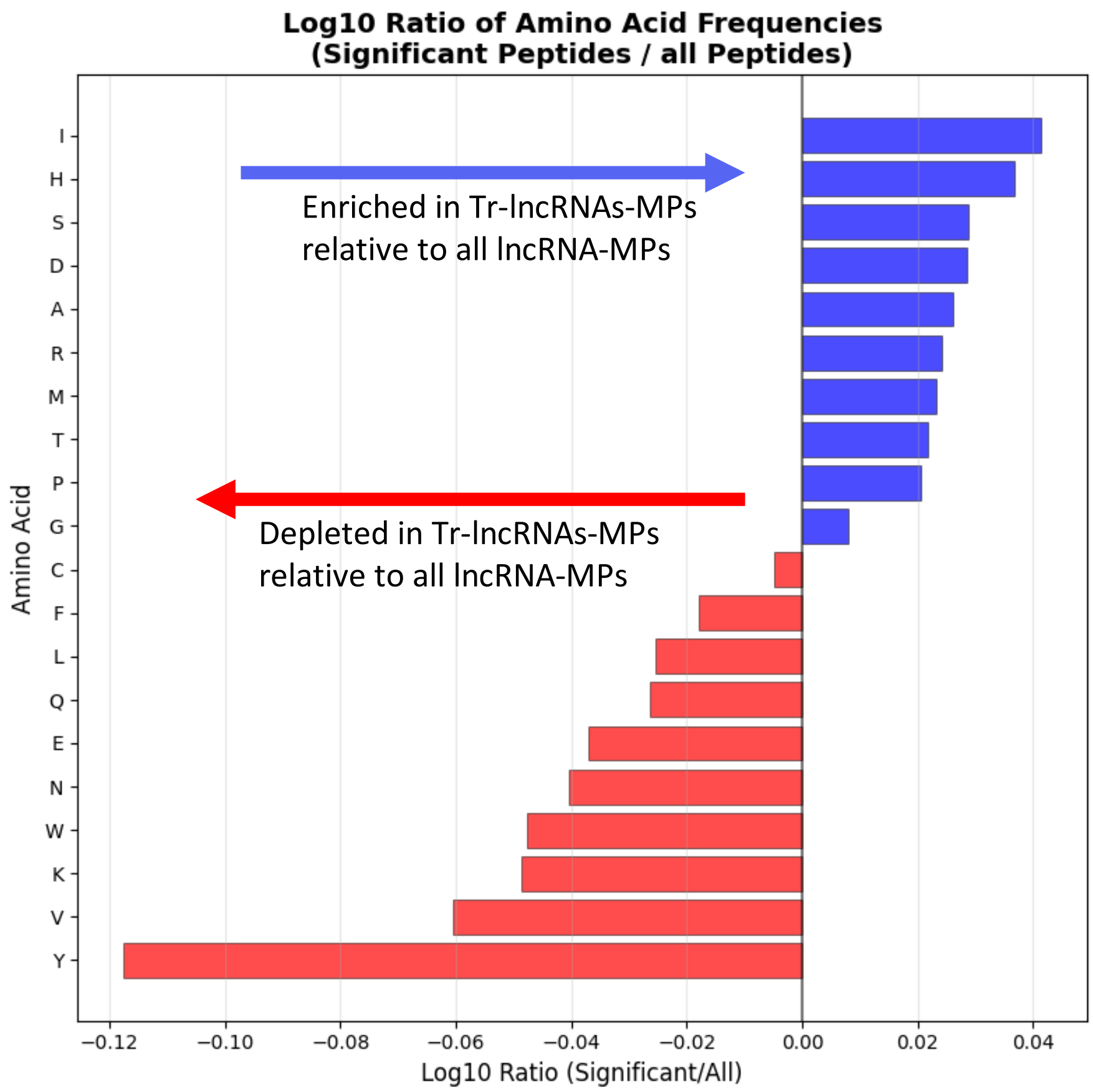

Fig. S3. *Amino acid (aa) composition indicating the differential use of each aa in the Tr-lncRNA-MPs (15,230 aa) relative to all-lncRNA-MPs (80,113 aa). The relative occurrence of Ile (I) is 1.4 and all values were converted to log10(Tr-lncRNAs to all-lncRNAs ratio) in the x-axis.*

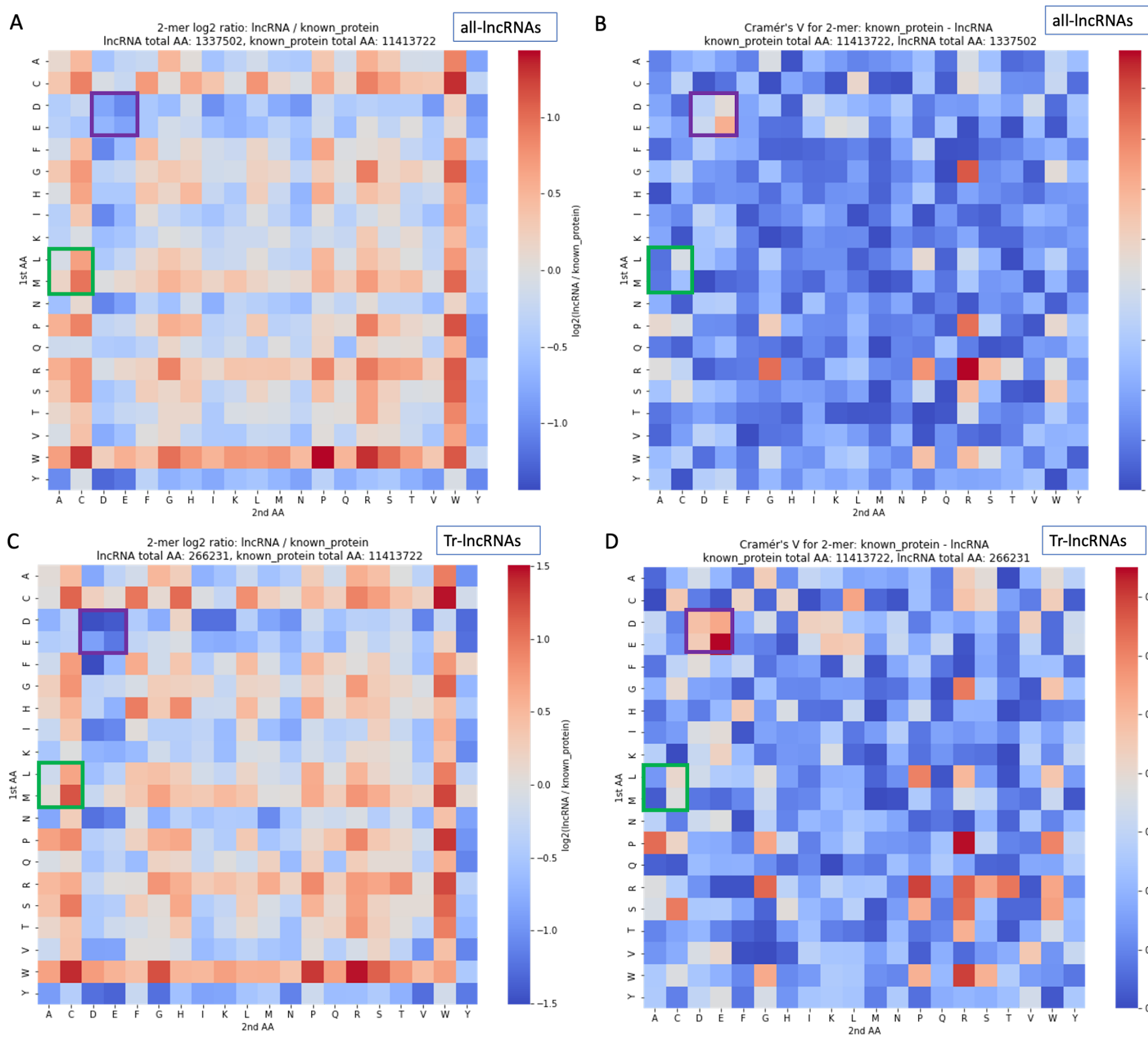

Fig. S4. *Matrices showing all 400 pairs of dipeptides for unfiltered lncRNA-MPs.* ***(A)*** *Heatmap of the* log_2*(ratio of lncRNAs to the human proteome).* ***(B)*** *A matrix showing the Cramer's V test for the association of the dipeptides in the human proteome (with 11.44 M aa) vs. the lncRNA-MPs (with 1.34M aa) and the strength of the associations. Values range from 0 to 1.0, where 0 indicates no association and 1.0 indicates maximal association.* ***(C)*** *A matrix showing all 400 pairs of dipeptides and their* log_2*(ratio of Tr-lncRNAs and human proteome).* ***(D)*** *A matrix showing the Cramer's V test for the association of the dipeptides in the human proteome vs. the Tr-lncRNAs and the strength of the associations. Note that Cramer's V values are positive, but B and D are scaled individually. The purple and green frames are for visualization purposes to highlight subtle differences in dipeptides across the different analyses.*

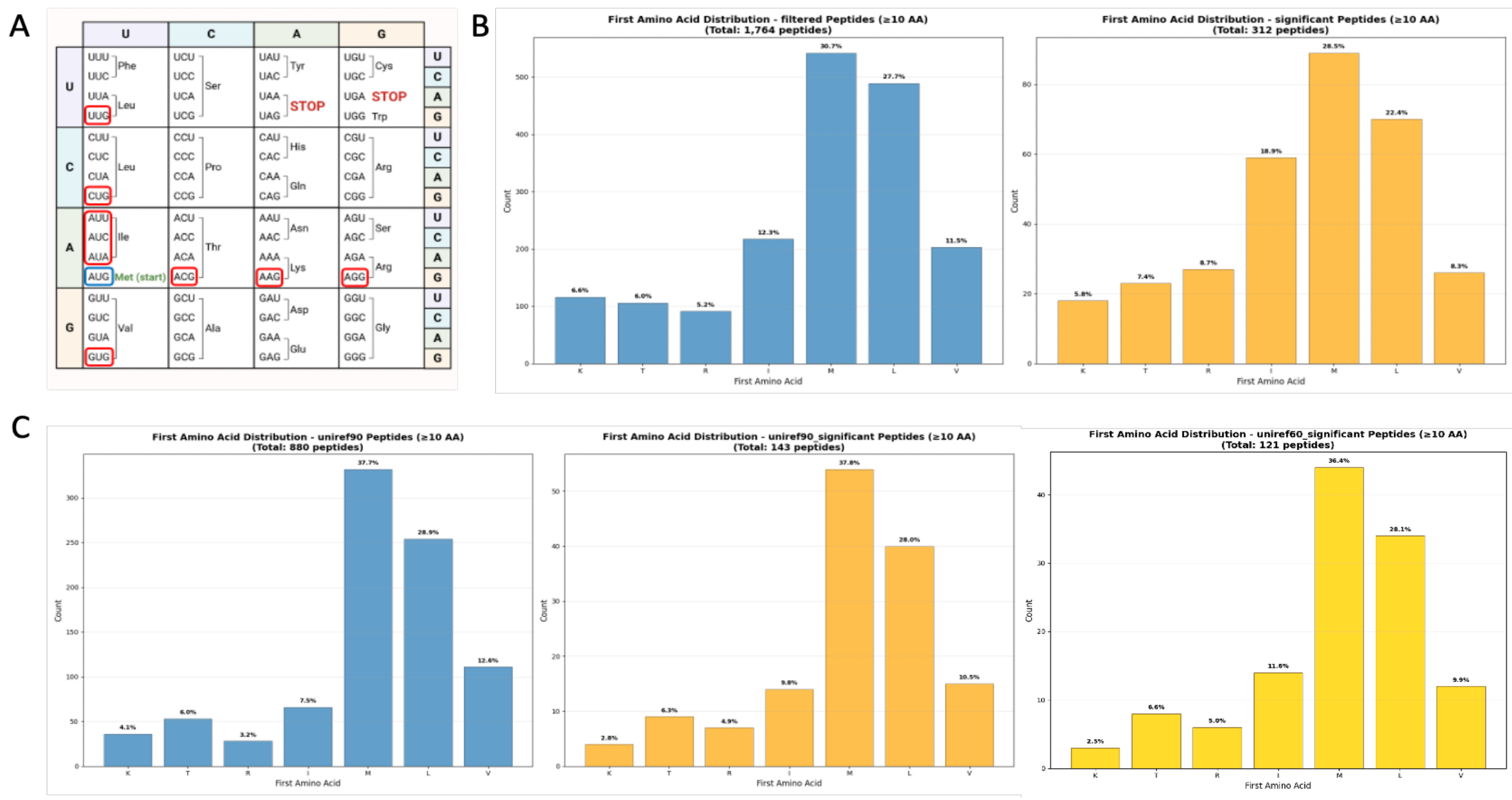

Fig. S5. *Analysis of MP initiation codon. The partition of the lncRNA-MPs by the first aa is shown for MPs >10 aa.* ***(A)*** *Codon table showing all near cognate AUG (Met) in red frames. The AUG Met is colored with a blue frame. Note that there are 2 and 3 codons that are 1-nt different from AUG for Leu and Ile, respectively.* ***(B)*** *Partition of the lncRNA-MPs (left, blue) and TR-lncRNA-MPs (orange, right). The number of peptides analyzed is indicated.* ***(C)*** *Reduced set by representatives according to Ref90 protocol (≥90% aa identity), resulting in 880 peptide representatives, while the Tr-lncRNA-MPs subset was reduced to 143 MPs. Further compression based on ≥60% identity reduction resulted in 121 peptides.*

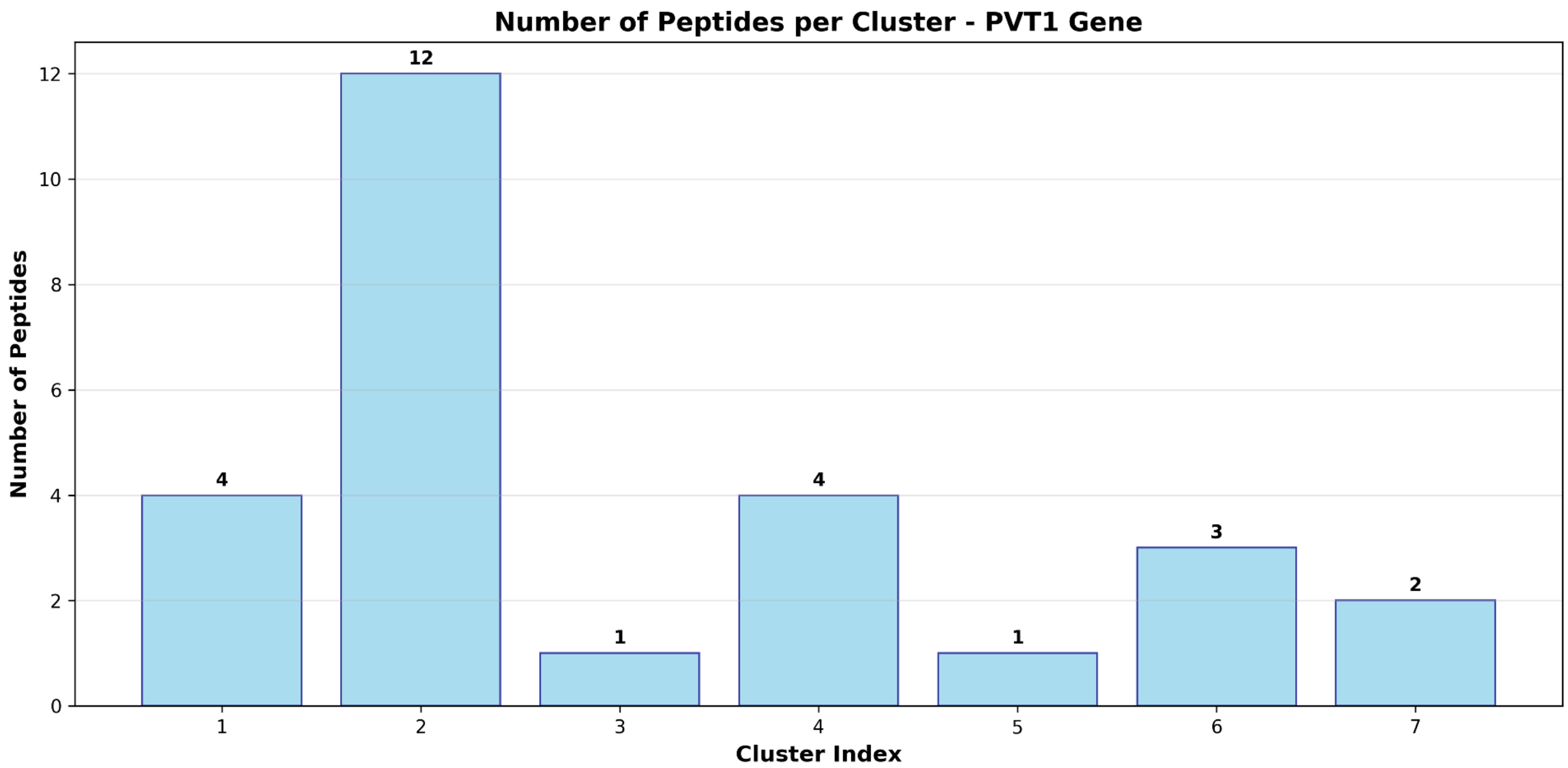

Fig. S6. *Sequence clustering of PTV1 with 27 high-quality MPs.* ***(A)*** *Clustering by sequence identity (>60%) of PTV1 (gene: ENSG00000249859.11). There are 7 peptide sequence-based clusters with non-overlapping sequences across the clusters.*

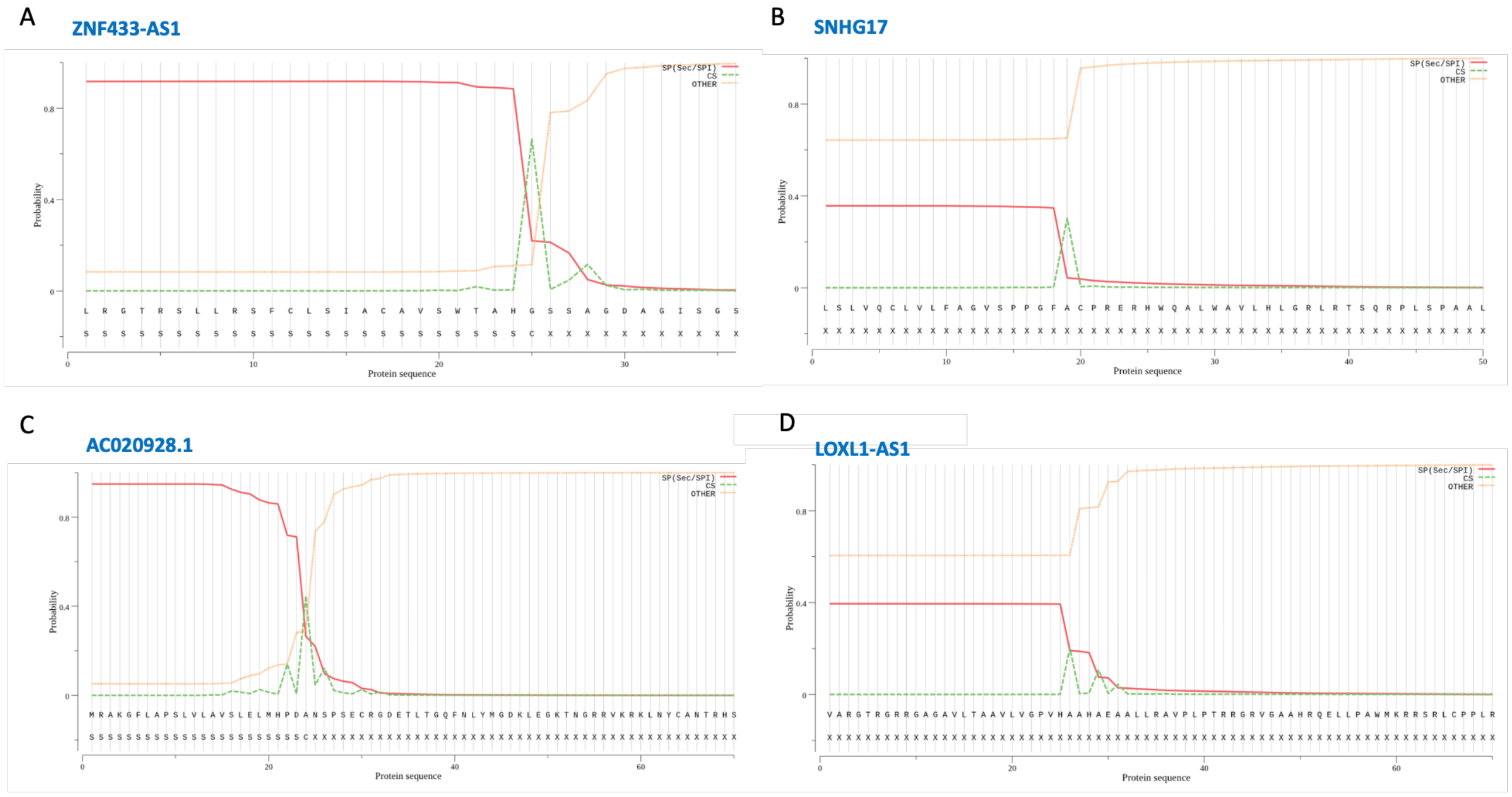

Fig. S7. *Signal P prediction for secretion potential of lncRNA-encoded MPs. There were 6 sequences (0.034%) that were shown to be a genuine candidate for having a signal peptide (SP).* ***(A)*** *ZNF433-AS1.* ***(B)*** *AC020928.1. These two genes are predicted with a strong SP prediction, with high Sec/SPI likelihood scores (>0.90).* ***(C)*** *SNHG17 and* ***(D)*** *LOXL1-AS1 displayed lower SP probabilities (>0.5, SignalP6.0). CS, cleavage site.*
